# Preventing tissue fibrosis by local biomaterials interfacing of specific cryptic extracellular matrix information

**DOI:** 10.1038/ncomms15509

**Published:** 2017-06-08

**Authors:** Christine-Maria Horejs, Jean-Philippe St-Pierre, Juha R. M. Ojala, Joseph A. M. Steele, Patricia Barros da Silva, Angela Rynne-Vidal, Stephanie A. Maynard, Catherine S. Hansel, Clara Rodríguez-Fernández, Manuel M. Mazo, Amanda Y. F. You, Alex J. Wang, Thomas von Erlach, Karl Tryggvason, Manuel López-Cabrera, Molly M. Stevens

**Affiliations:** 1 Department of Materials, Imperial College London, Exhibition Road, London SW7 2AZ, UK; 2 Department of Bioengineering, Imperial College London, Exhibition Road, London SW7 2AZ, UK; 3 Institute of Biomedical Engineering, Imperial College London, Exhibition Road, London SW7 2AZ, UK; 4 Department of Medical Biochemistry and Biophysics, Karolinska Institutet, Scheeles väg 2, Stockholm 17177, Sweden; 5 Centro de Biología Molecular Severo Ochoa, CSIC, Universidad Autónoma de Madrid, Campus de Cantoblanco, 28049 Madrid, Spain; 6 Department of Chemistry, Imperial College London, Imperial College Road, London SW7 2AZ, UK; 7 Cardiovascular and Metabolic Disorders Program, Duke-NUS, 8 College Road, Singapore 169857, Singapore

## Abstract

Matrix metalloproteinases (MMPs) contribute to the breakdown of tissue structures such as the basement membrane, promoting tissue fibrosis. Here we developed an electrospun membrane biofunctionalized with a fragment of the laminin β1-chain to modulate the expression of MMP2 in this context. We demonstrate that interfacing of the β1-fragment with the mesothelium of the peritoneal membrane via a biomaterial abrogates the release of active MMP2 in response to transforming growth factor β1 and rescues tissue integrity *ex vivo* and *in vivo* in a mouse model of peritoneal fibrosis. Importantly, our data demonstrate that the membrane inhibits MMP2 expression. Changes in the expression of epithelial-to-mesenchymal transition (EMT)-related molecules further point towards a contribution of the modulation of EMT. Biomaterial-based presentation of regulatory basement membrane signals directly addresses limitations of current therapeutic approaches by enabling a localized and specific method to counteract MMP2 release applicable to a broad range of therapeutic targets.

## Abstract (web summary)

Matrix metalloproteinase enzymes are widely known to contribute to the breakdown of tissues. Here, the authors utilise a biomaterial substrate functionalised with a laminin fragment to modulate the expression of these enzymes, subsequently controlling the integrity of tissue.

Tissue fibrosis is a major cause of organ dysfunction triggered by either acute or chronic inflammation that can affect every tissue and organ in the body^1^. To date, there are limited therapeutic treatments available to slow the progression of fibrosis. This is largely due to the heterogeneous evolution of the disease in different tissues, the limited understanding of its pathogenesis and the complexity of signalling pathways involved. However, increased levels of matrix metalloproteinases (MMPs) that degrade the basement membrane have been detected in a variety of fibrotic tissues, and it has been demonstrated that processes related to the epithelial-to-mesenchymal transition (EMT)^2^,^3^ can contribute to the accumulation of α-smooth muscle actin (α-SMA) expressing myofibroblasts in certain fibrotic phenotypes^4^,^5^,^6^,^7^, for example, in peritoneal, pulmonary, liver and kidney fibrosis, while fate-mapping studies identified diverse sources of myofibroblasts depending on methodology, tissue type and experimental model^8^,^9^,^10^. The activation of EMT transcription factors (EMT-TFs) such as snail or slug and the consequent downregulation of the key epithelial proteins such as E-cadherin or tight junction protein 1 (ZO-1) have been defined as EMT hallmarks^11^. Although the significance of EMT pathways in tissue fibrosis is still under debate^12^, the proteolytic breakdown of the basement membrane by MMPs has been shown to be a key component of fibrotic pathologies^2^,^13^,^14^,^15^,^16^,^17^ (Fig. 1a).

Interestingly, it has recently been demonstrated that conditional deletion of EMT-TFs is an effective strategy to inhibit renal fibrosis in a mouse model^18^,^19^. Moreover, MMP-knockout mice have confirmed a correlation between EMT-TFs and MMPs that leads to breaching of the basement membrane and facilitates the cell transition to a migratory phenotype^20^,^21^. The transition of mesothelial cells in the course of peritoneal fibrosis has also been shown to contribute significantly to the observed accumulation of myofibroblasts in the tissue interstitium in both animal models and patient-derived tissues^22^,^23^,^24^. Targeting EMT in this context has been suggested as a promising strategy to inhibit fibrosis^25^,^26^,^27^. Therefore, reducing MMP activity offers an opportunity to prevent basement membrane degradation and EMT, among other effects. However, soluble MMP inhibitors, along with other therapeutics aimed at interfering with EMT pathways, have proved difficult to translate to the clinic owing to detrimental side effects caused by a limited therapeutic window and off-target effects^28^.

We have recently reported a cryptic laminin fragment generated by MMP2 processing of the laminin β1-chain that modulates the expression of *Mmp2* and EMT-associated molecules in embryonic stem cells and that interacts with α3β1-integrin^29^. Integrins are involved in MMP and cell phenotype regulation, and the interaction of certain basement membrane domains with specific integrins is subject to constant alterations during EMT^30^,^31^. Specifically, α3-integrin has been shown to play a role in pulmonary fibrosis, where EMT is initiated through an integrin-dependent association of tyrosine-phosphorylated β-catenin and p-smad2 (ref. 32). We therefore hypothesize that the regulation of MMP2 expression and EMT-related molecules by the laminin β1-fragment can prevent basement membrane degradation, with potential therapeutic benefits for tissue fibrosis.

As ∼45% of all deaths in the western world can be attributed to organ or tissue fibrosis^33^ and because of the aforementioned limitations of antifibrotic therapies, there is an urgent need for developing localized and specific strategies to prevent fibrosis. Purcell *et al*.^34^ have recently demonstrated the benefits of localized delivery of tissue inhibitors of MMPs to address extracellular matrix degradation. The work presented here builds on this concept with the advantage that it aims to specifically have an impact on the expression of MMP2 and thus basement membrane breaching and ultimately fibrosis (Fig. 1b). Our strategy aims to interface a cryptic recombinant laminin fragment with epithelial tissue via localized biomaterial delivery to circumvent off-target effects associated with the use of soluble compounds. This is achieved by the immobilization of the fragment on a flexible electrospun poly(ɛ-caprolactone) (PCL) membrane^35^ via the deposition of a polydopamine (pDA) coating^36^. This simple approach provides versatility as it permits the immobilization of large proteins and other compounds on a range of substrates in a way that retains their bioactivity^37^,^38^ and is amenable to increased biological complexity towards further control of MMP expression, EMT-related events and other processes that cause the accumulation of myofibroblasts during fibrosis. In its current form, the material intervention presented here is suited for short-term interventions for relatively small tissue areas, such as is required for the prevention of peritoneal adhesions following abdominal surgeries, including minimization of local effects following the insertion of catheters for peritoneal dialysis.

Here we characterize the functionalization of an electrospun biomaterial with the recombinant laminin β1-fragment and we show that it can be directly interfaced with epithelial cells and tissues to inhibit MMP2 expression and activity, both *in vitro* and *in vivo*. We demonstrate how interaction of the functionalized synthetic membrane with peritoneal tissue decreases matrix deposition associated with fibrosis and rescues the peritoneal basement membrane in a mouse model of TGFβ-induced peritoneal fibrosis. Furthermore, we propose a signalling mechanism by which the laminin β1-fragment acts downstream of α3β1-integrin in epithelial cells, after it is released from the basement membrane.

## Results

### Electrospun membrane functionalization with laminin fragment

**Figure 1:**
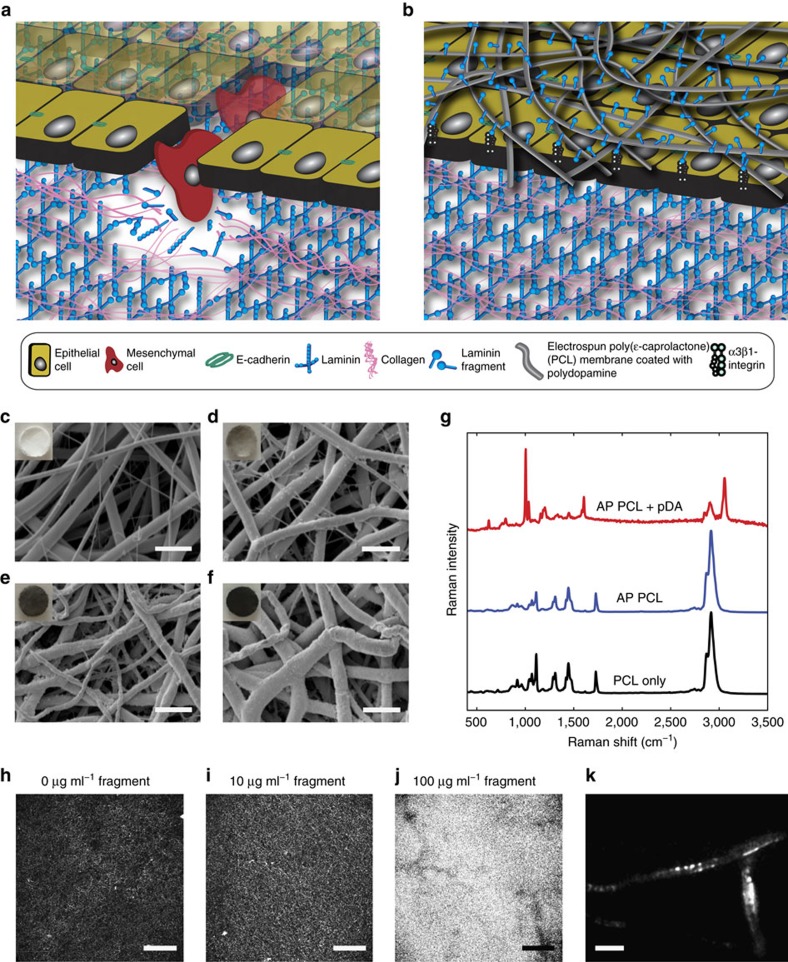
Electrospun membrane functionalized with laminin fragment. (**a**) Schematic representation of basement membrane breakdown and biomaterials approach. Cells undergo phenotype alterations, accompanied by an upregulation of active MMPs that results in the degradation of the basement membrane. The action of active MMPs exposes cryptic ECM information such as the laminin β1-fragment. (**b**) Schematic representation of the proposed material developed to prevent increased MMP activity as a response to TGFβ1. A PCL membrane is functionalized with a fragment of the laminin β1-chain via a pDA coating and directly interfaced with an epithelial cell layer. (**c**–**f**) Scanning electron micrographs of air plasma-treated (AP) electrospun PCL membranes (**c**) before and (**d**) after 1 h, (**e**) 4 h and (**f**) 24 h incubation in a 2 mg ml^−1^ dopamine solution to apply a pDA coating. Scale bars, 10 μm; magnification: × 1,000. Insets contain photographs of the membranes. (**g**) Raman spectra of (black trace) as-made membranes, as well as AP membranes (blue trace) before and (red trace) after pDA coating (4 h). (**h**–**k**) Confocal images of membranes coated with pDA for 4 h after incubation with (**h**) 0 μg ml^−1^, (**i**) 10 μg ml^−1^ and (**j**) 100 μg ml^−1^ fluorescein isothiocyanate-labelled laminin β1-fragment for 8 h at 37 °C. Scale bars, 150 μm. (**k**) Total internal reflection fluorescence image of pDA-coated electrospun PCL fibres after incubation with 10 μg ml^−1^ fluorescein isothiocyanate-labelled laminin β1-fragment for 8 h at 37 °C. Scale bar, 5 μm.

Electrospinning has been used extensively to produce flexible nonwoven membranes composed of fibres made from a range of natural or synthetic polymers. While some biological activity can be inferred from the use of natural polymers as a base material, synthetic polymers offer increased control over the structural and mechanical properties of the material at the cost of biological functionality^39^. Multiple biofunctionalization approaches have been developed to modify electrospun scaffolds and provide specific and localized biological cues to interfacing cells and tissues^35^. One such approach that has recently generated interest in the area of biomaterial functionalization involves deposition of a pDA coating via an oxidative self-polymerization process at slightly basic pH to mimic mussel adhesion proteins^36^. This approach provides increased versatility in that it can easily be implemented to immobilize large peptides and proteins on a wide range of substrates and in a way that retains their bioactivity at least in part. The interaction between proteins and the pDA coating has been shown to involve Schiff base and Michael addition reactions between amine and thiol moieties and the catechol groups in the coating^37^,^38^. In this work, electrospun PCL membranes were prepared such that ∼0.5–2.0 μm diameter fibres formed randomly oriented nonwoven mats, visualized by scanning electron microscopy (SEM; Fig. 1c–f and Supplementary Fig. 1). The pDA coating of the PCL fibres was optimized to immobilize a uniform, high density of cryptic extracellular matrix (ECM) signals to interfacing epithelial cells. Plasma treatment was performed on the PCL membranes to render them hydrophilic and to produce a more uniform pDA coating and subsequent protein functionalization. This step did not affect the overall morphology of the fibres (Supplementary Fig. 2). Time-dependent pDA coating of the membrane showed that, after 1 h incubation in the dopamine solution, the PCL fibres exhibited a roughened surface but did not appear to be fully covered as some areas were smooth and featureless as with the uncoated PCL fibres (Fig. 1d and Supplementary Fig. 1). After 4 h incubation, the PCL fibres appeared completely coated (Fig. 1e and Supplementary Fig. 1). The coating became fragmented when the PCL membranes were incubated for 24 h in the dopamine solution (Fig. 1f and Supplementary Fig. 1). Over increasing incubation time, the colour of the PCL membranes changed from white to grey of increased intensity (Fig. 1c–f insets) and the average fibre diameter increased with incubation time in the dopamine solution (0 h: 1.26±0.49 μm; 1 h: 1.39±0.39 μm; 4 h: 1.76±0.23 μm; 24 h: 1.70±0.35 μm), suggesting pDA deposition.

Deposition of the pDA coating was further confirmed with a characterization of the chemical composition of the membranes by Raman spectroscopy (Fig. 1g). Raman spectra for PCL membranes with the pDA coating exhibited peaks corresponding to aromatic ring breathing (1,010 cm^−1^), C=C vibrations (1,609 cm^−1^) and aromatic ring CH vibrations (3,057 cm^−1^; refs 40, 41). No major differences were observed in the Raman spectra of uncoated PCL membranes before and after plasma treatment.

The application of a pDA coating on electrospun PCL membranes led to a statistically significant increase in BSA (model protein) immobilization after 4 and 24 h incubations in the dopamine solution (Supplementary Fig. 3). No significant difference was observed between the two longer incubation times. On the basis of the BSA-binding data and morphological observations obtained with SEM, 4 h incubation in the dopamine solution was deemed optimal and was used for all further experiments. The adsorption of the laminin fragment was verified by incubating pDA-coated PCL membranes with different concentrations of the fluorescein isothiocyanate (FITC)-labelled protein. A concentration-dependent increase in the fluorescence signal was observed qualitatively on the membranes by confocal microscopy (Fig. 1h–j). Total internal reflection fluorescence microscopy confirmed the presence of FITC-labelled fragment on the surfaces of individual pDA-coated electrospun PCL fibres (Fig. 1k). Using the quantitative protein-binding assay described for the evaluation of BSA binding, it was determined that 1.86±0.31 μg of the fragment was immobilized during the 8 h incubation representing 93% of the theoretical initial protein content in the solution according to the optimized membrane functionalization protocol for *in vitro*, *ex vivo* and *in vivo* experiments.

### Functionalized membrane interferes with MMP2 expression

**Figure 2:**
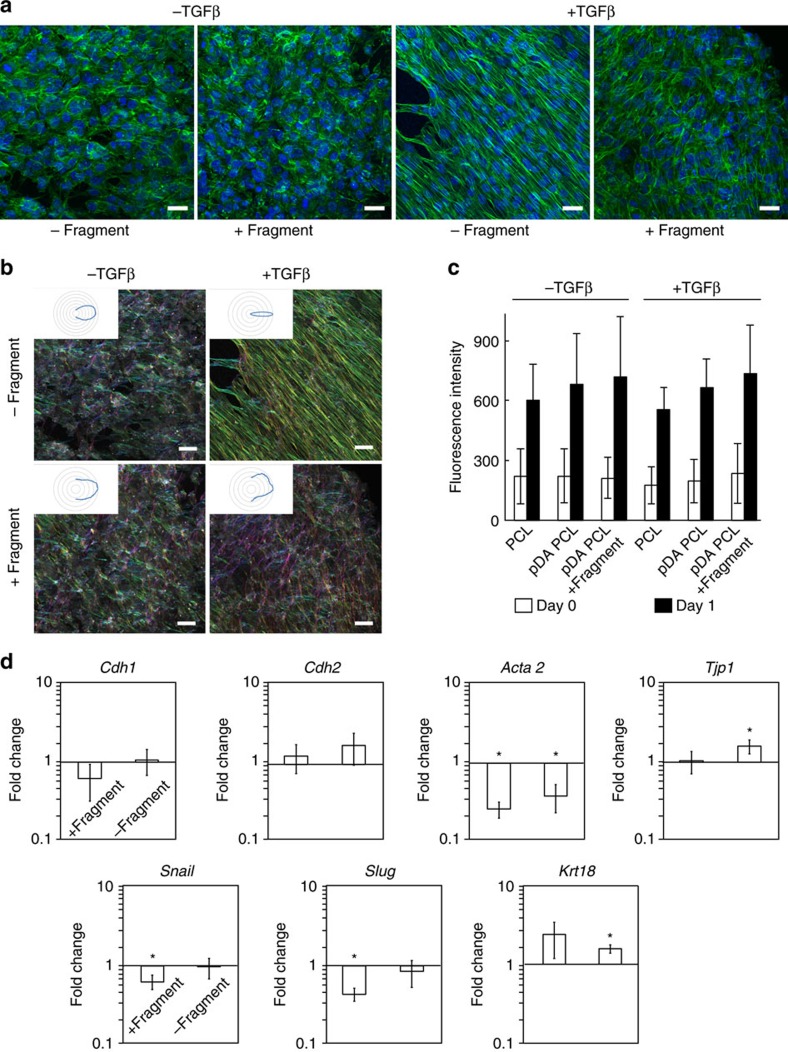
Mouse mammary gland epithelial cells cultured on membranes. (**a**) Representative immunofluorescence images (phalloidin staining, green; DAPI staining, blue) of NMuMG cells cultured directly on the membranes without (-fragment) and with (+fragment) immobilized recombinant laminin β1-fragment, in the absence (−TGFβ) and presence (+TGFβ) of TGFβ1 for 24 h. Scale bars, 25 μm. (**b**) Colour survey of the orientation of actin fibres obtained by phalloidin staining and radar plots of the orientation distributions in insets. Colour gradient: red=90°, pink-blue=+45°, yellow-green=−45°. (**c**) Metabolic activity of NMuMG cells cultured on electrospun PCL membranes without pDA coating (PCL), with pDA (pDA PCL) and with pDA and functionalized with laminin fragment (pDA PCL+fragment) as measured with an Alamar blue assay at time points 0 h (white bars) and 24 h (black bars). The metabolic activity is normalized to values obtained for acellular membranes. Data shown as mean±s.d. (**d**) Gene expression profile of NMuMG cells on pDA-coated PCL membranes with (+fragment) and without (−fragment) immobilized recombinant laminin β1-fragment. *y* axis: log(2^(−ΔΔct)^). All data are normalized to GAPDH and NMuMG cells cultured on TCP. Data shown as mean±s.e.m. *P<0.05.

TGFβ1 treatment of normal mouse mammary gland epithelial (NMuMG) cells has been extensively used as a model to study TGFβ-induced EMT changes in epithelial cells *in vitro*^42^. We employed this model to implement a first demonstration of the ability of the laminin fragment-functionalized membrane to mitigate the changes associated with TGFβ-induced EMT and MMP2 expression. First, we evaluated the biocompatibility of the material and validated the *in vitro* model of EMT when carried out on the membranes. NMuMG cells adhered to electrospun PCL membranes and exhibited anticipated morphological changes characterized by cytoskeletal reorganization from a cortical arrangement to elongated actin fibres in response to TGFβ1 treatment for 24 h when cultured on the electrospun PCL membranes (Fig. 2a). It was noticed that a small number of cells in contact with the non-functionalized membrane maintained a more cobble stone-like morphology despite the TGFβ1 treatment (Supplementary Fig. 4). We also show the effect of membrane functionalization with the laminin fragment on the extent of cytoskeletal reorganization (Fig. 2a). Of note, the cells in contact with the membrane maintained the cobble stone-like morphology typical of epithelial cells, while some cells on top of these exhibited an elongated morphology (Supplementary Fig. 4). It is possible that the elongated cells did not exhibit extensive contact with the functionalized membranes upon initiation of the TGFβ1 treatment, a situation that is likely a result of the *in vitro* model and would not be a major concern when interfacing the membrane with epithelial tissue. We further demonstrate that changes in the degree of orientation of actin fibres occurring in response to TGFβ1 treatment were abrogated by the fragment-functionalized membranes (Fig. 2b). This is confirmed by evaluation of the distribution of actin fibre orientation in confocal images, which clearly indicates a broader spectrum of orientations in cells cultured on functionalized than non-functionalized membranes (Fig. 2b insets).

Comparable metabolic activities were measured for NMuMG cells cultured on pDA-coated electrospun PCL membranes with and without fragment functionalization, as well as uncoated PCL membranes after 1 day (Fig. 2c). Taken together, these results suggest that the pDA coating and the laminin fragment do not adversely affect the biocompatibility of the material. While many studies have reported altered cell proliferation on pDA-coated materials^43^,^44^, the results presented here suggest that at least metabolic activity is not affected within the system studied and for the time frame investigated. Moreover, gene expression of *Cdh1*, *Tjp1* and *Krt18* in NMuMG cells cultured on the pDA-coated electrospun PCL membranes with and without fragment functionalization was not altered or increased relative to normal culture conditions on tissue culture plastic (TCP). In addition, mesenchymal genes *Snail*, *Slug* and *Acta2* were significantly downregulated on the fragment-functionalized membranes (Fig. 2d), while cells expressed *Cdh1* as expected in both conditions.

**Figure 3:**
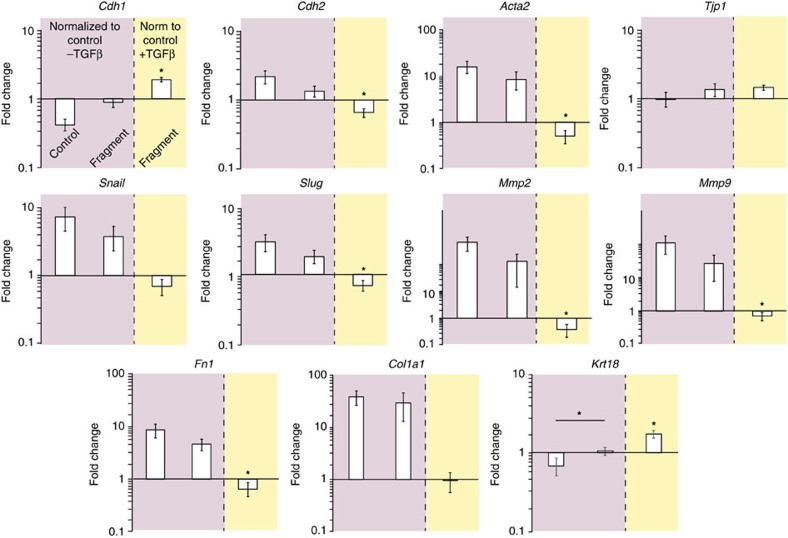
Changes in EMT. Gene expression profile of NMuMG cells undergoing TGFβ1-induced EMT at 24 h post treatment on membranes without (control) and with the laminin β1-fragment (fragment). *y* axis: log(2^(−ΔΔct)^). All data are normalized to GAPDH and NMuMG cells cultured on non-functionalized membranes without TGFβ1 treatment (pink) or with TGFβ1 treatment (yellow). Data shown as mean±s.e.m. *P<0.05.

EMT was initiated in NMuMG cells cultured on non-functionalized membranes by incubation with TGFβ1 for 24 h. *Cdh1* and *Krt18* were downregulated compared to cells that did not receive the TGFβ1 treatment, while *Tjp1* did not exhibit a significant change in expression (Fig. 3). Genes associated with EMT (*Cdh2, Acta2, Mmp2, Mmp9, Snail, Slug, Col1a1* and *Fn1*) were upregulated (Fig. 3). Of note, NMuMG cells cultured on the functionalized membranes and treated with TGFβ1 for 24 h retained basal expression levels for genes associated with the epithelial phenotype, representing a significant difference compared to cells cultured on non-functionalized membranes and treated with TGFβ1 for *Cdh1* (Fig. 3). Correspondingly, a number of genes upregulated during EMT (*Cdh2, Acta2, Mmp2, Mmp9, Slug, Fn1*) were significantly downregulated when NMuMG cells were cultured on the functionalized membrane compared to the control material (Fig. 3). These data suggest that interfacing the laminin β1-fragment with NMuMG cells can modulate a number of EMT-related genes in the presence of TGFβ1.

**Figure 4:**
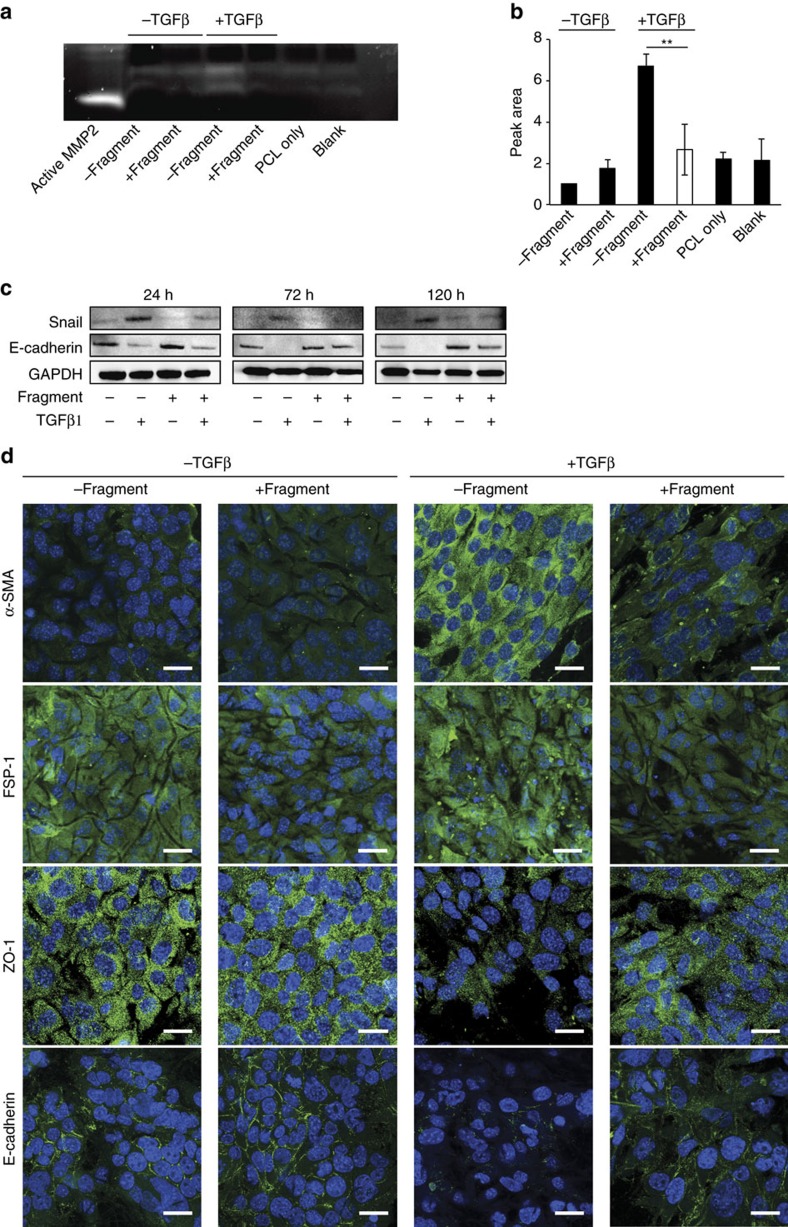
Changes in MMP2 and EMT. (**a**) Active MMP2 levels assessed by gelatin zymography of conditioned media of NMuMG cells cultured on membranes without (−fragment) and with laminin β1-fragment (+fragment), with or without TGFβ1 treatment for 24 h. White bands show degradation of the gelatin gel by pro- and active MMP2. ‘Active MMP2' refers to the recombinant-active enzyme (positive control). ‘Blank' refers to the background from the medium. ‘PCL only' refers to acellular membranes. (**b**) Densitometric quantification of zymograms from three independent experiments. Peak area was normalized to non-functionalized membranes without TGFβ1 treatment, **P<0.005. (**c**) Representative immunoblots of NMuMG cells cultured on membranes without and with laminin fragment, undergoing TGFβ1-induced EMT, 24, 72 and 120 h after induction. (**d**) Representative immunofluorescence images of α-SMA, FSP-1, ZO-1 and E-cadherin expression by NMuMG cells cultured on membranes with and without immobilized laminin β1-fragment in the presence or absence of TGFβ1 (antibody labelling, green; DAPI staining, blue). Scale bars, 20 μm.

In agreement with gene expression data, the levels of active MMP2 measured in conditioned media by gelatin zymography were significantly lower in NMuMG cells cultured on membranes functionalized with the laminin fragment than on non-functionalized membranes following TGFβ1 treatment (Fig. 4a and whole gel in Supplementary Fig. 5). Interestingly, the activity levels were maintained at the baseline level observed for cells that were not treated with TGFβ1. Densitometric evaluation of zymography from independent experiments confirmed that this effect of the laminin fragment on active MMP2 levels was significant (Fig. 4b). To further show specificity of the effect of the fragment functionalization on MMP2 activity levels, the membranes were coated with different recombinant laminin isoforms (murine laminin-111 and human laminin-521) and MMP2 activity in NMuMG cells treated with TGFβ1 was assessed by gelatin zymography. However, no changes in MMP2 activity were observed in cells interacting with laminin-111 or laminin-521 (Supplementary Fig. 6). The effect of the fragment functionalization on MMP2 expression was further tested using primary mouse peritoneal mesothelial cells, and the same decrease in MMP2 activity levels could be measured by gelatin zymography as with NMuMG cells, validating the effect of the laminin fragment on MMP2 in a cell-type relevant to the intended application (Supplementary Fig. 7).

The fragment functionalization was also observed to substantially prevent the decrease in E-cadherin expression associated with TGFβ1 treatment, as well as the increase in snail expression (Fig. 4c and Supplementary Figs 5 and 8). The effect of the fragment-functionalized membrane was maintained for at least 5 days (Fig. 4c). Further, NMuMG cells cultured on fragment-functionalized membranes exhibited visible differences in E-cadherin, ZO-1, α-SMA, FSP-1 and N-cadherin protein levels, as shown by immunofluorescence labelling (Fig. 4d and Supplementary Fig. 9). However, seeding NMuMG cells on laminin fragment-functionalized membranes did not significantly alter collagen type I gene expression and collagen deposition in response to treatment with TGFβ1 (Fig. 3 and Supplementary Fig. 10).

Taken together, these results suggest that the fragment-functionalized membranes can interact with epithelial cells (NMuMG cells and primary mouse peritoneal mesothelial cells) cultured *in vitro* and prevent many of the phenotypic changes associated with TGFβ1-induced EMT. While the gene expression data show a modest but significant effect of the fragment on the expression of some EMT-related genes compared to cells cultured on non-functionalized membranes, protein-level expression and activity measurements suggest an important modulation of EMT. Indeed, the downregulation in *Mmp2* gene expression in TGFβ1-treated cells interacting with the fragment is correlated with levels of released active MMP2 that are comparable to those of cultures that were not treated with TGFβ1.

This is of major importance to the potential therapeutic applications of this material, as inhibition of the pathological effects of MMPs has been extensively pursued for a number of biomedical applications including those resulting from chronic inflammation^28^.

### Laminin fragment interacts with α3β1-integrin and alters EMT

**Figure 5:**
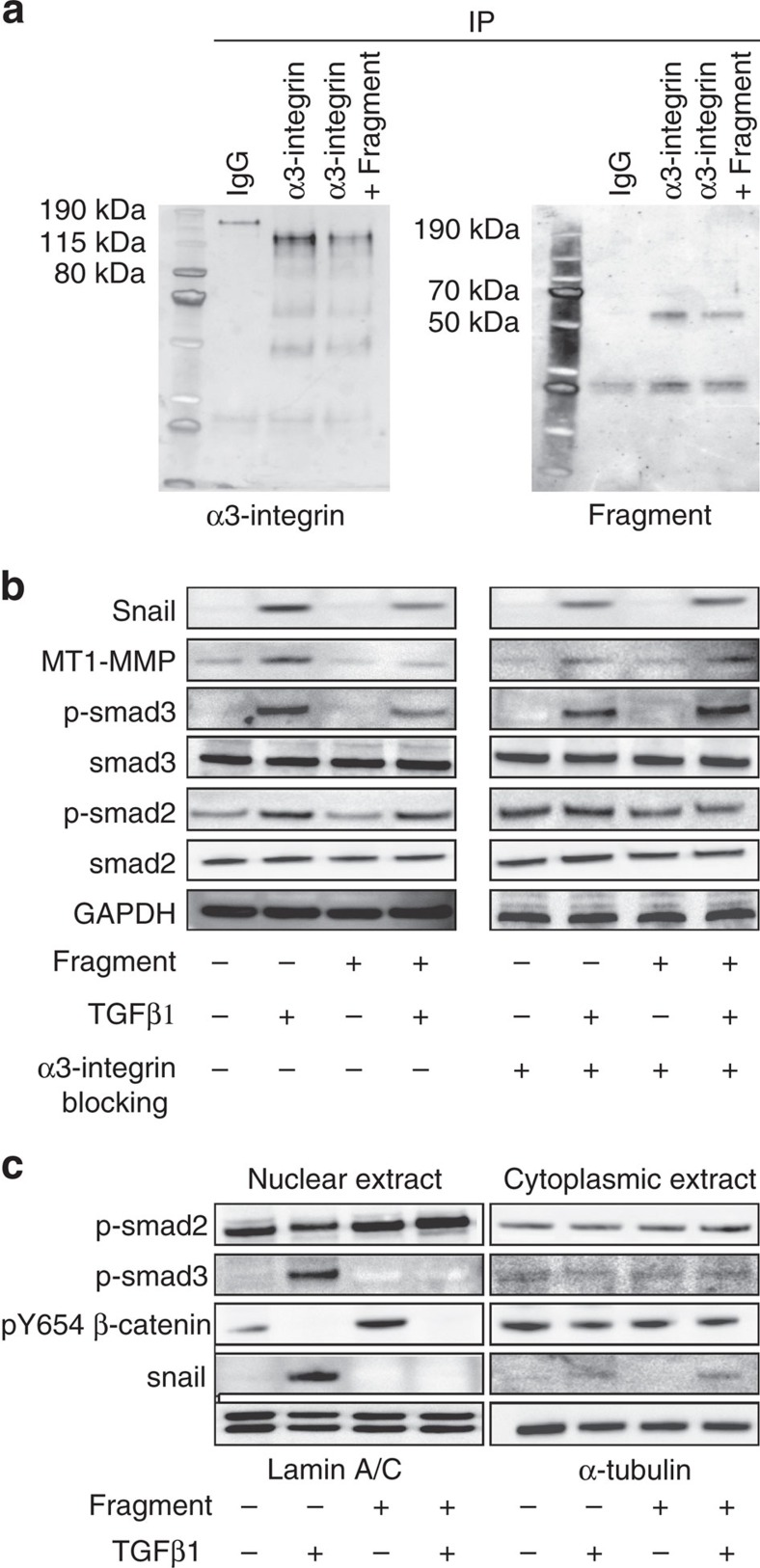
Fragment binds α3-integrin and alters cell signalling. (**a**) NMuMG cell extracts treated with or without soluble laminin β1-fragment were immunoprecipitated using an α3-integrin antibody and immunoblotted with an antibody against α3-integrin or the laminin β1-fragment. IgG was used as control. (**b**) Representative immunoblots of NMuMG cells treated with or without laminin β1-fragment, with or without TGFβ1 and with or without a specific α3-integrin blocking antibody for 24 h. (**c**) Nuclear and cytoplasmic extracts of NMuMG cells treated with or without β1-fragment and with or without TGFβ1 for 24 h.

In order to shed light on the pathways influenced by interaction of the laminin β1-fragment with NMuMG cells, we first demonstrated the specific interaction of the fragment with α3-integrin by immunoprecipitation (Fig. 5a). While we have demonstrated in our previous work that the laminin β1-fragment interacts with α3β1-integrin^25^, the immunoprecipitation data shown here further suggest that the fragment interacts, directly or indirectly, with α3-integrin, but not with α6-integrin (data not shown), in NMuMG cells. To elucidate the pathways affected by binding of the laminin β1-fragment to α3-integrin, we cultured NMuMG cells in the absence or presence of TGFβ1 for 24 h and investigated changes in smad-dependent EMT pathways following treatment with 10 μM soluble laminin β1-fragment. TGFβ-induced phosphorylation and subsequent nuclear translocation of smad2 and smad3 during EMT are well-established events that lead to the modulation of the expression of a number of genes, including *Snail*^45^. As expected, NMuMG cells treated with TGFβ1 in absence of the laminin β1-fragment exhibited an upregulation of p-smad2, p-smad3, snail and MT1-MMP protein levels^45^ (Fig. 5b and Supplementary Fig. 11). Treatment with the laminin β1-fragment mitigated p-smad3, snail and MT1-MMP upregulations, but did not exhibit an effect on p-smad2 (Fig. 5b and Supplementary Fig. 11). Specific blocking of α3-integrin with a blocking antibody rescued the upregulation of p-smad3, snail and MT1-MMP, in the presence of the laminin β1-fragment (Fig. 5b). Further, nuclear extracts revealed that p-smad3 and snail are not located in the nucleus of cells interacting with the laminin β1-fragment, whereas nuclear p-smad2 levels do not appear to be affected by any of the treatment conditions (Fig. 5c).

These data show that in cells treated with TGFβ1, the interaction between the laminin β1-fragment and α3-integrin leads to changes in total protein phosphorylation levels and nuclear localization of both p-smad3 and snail, but not p-smad2. Moreover, nuclear tyrosine 654-phosphorylated β-catenin was not detected in cells cultured in the presence of both the laminin β1-fragment and TGFβ1, while it was detected in cells that were not treated with TGFβ1 (Fig. 5c)^32^. The inhibitory effect of the laminin β1-fragment on MT1-MMP protein levels is also interesting as this protease has been associated with basement membrane degradation and is also known to activate MMP2 (refs 20, 21, 46, 47).

### Functionalized membrane rescues peritoneal mesothelium

**Figure 6:**
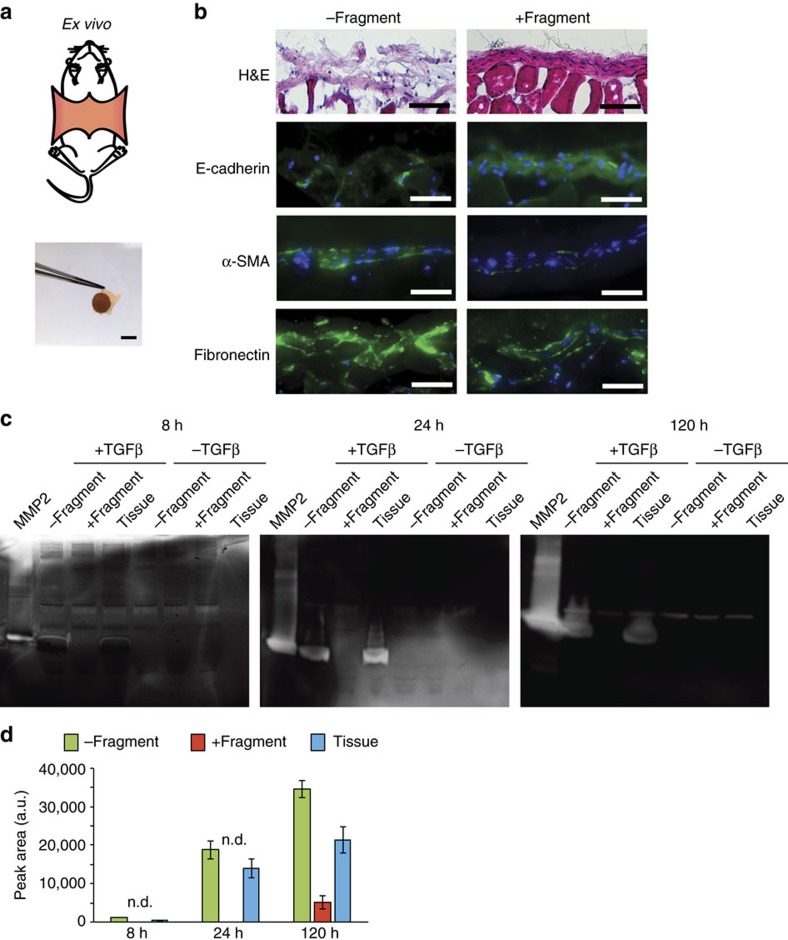
Membranes interfaced with mouse peritoneal tissue explants. (**a**) *Ex vivo* study: the mesothelium was explanted from mice by removing the skin layer and excising the inner abdominal wall. Membranes were directly interfaced with the mesothelium and cultured *ex vivo* for 24 h. Scale bar, 5 mm. (**b**) Representative haematoxylin and eosin (H&E) and immunohistofluoresence images of mouse peritoneal tissue explants interfaced with membranes with or without the laminin β1-fragment after 24 h treatment with TGFβ1, stained for E-cadherin, α-SMA and fibronectin (antibody labelling, green; DAPI, blue). Scale bars, 100 μm. (**c**) Representative gelatin zymographs of conditioned media of mouse peritoneal tissue explants interfaced with membranes with or without the laminin β1-fragment taken 8, 24 and 120 h after initiation of treatment with TGFβ1. ‘MMP2' refers to active MMP2 control; ‘Tissue' refers to untreated tissue control. (**d**) Corresponding densitometric semi-quantitative analysis of zymograms shown in **c**. Data shown as peak area. *N*=3. Data shown as mean±s.e.m. n.d., not detectable refers to no bands on the gel.

The clinical applicability of a biofunctionalized electrospun membrane such as the one developed in this work relies on the ability to successfully promote interactions between epithelial tissue and the fragment to control cellular responses to TGFβ1. The peritoneal membrane is covered by a monolayer of mesothelial cells that can undergo a mesothelial-to-mesenchymal transition (MMT), which is a type of EMT that contributes significantly to peritoneal fibrosis upon chronic inflammatory insults^23^. Moreover, in patient-derived peritoneal effluents, elevated MMP2 levels have been correlated with peritoneal injury and fibrosis^48^. Peritoneal fibrosis is a common response to peritoneal dialysis, chemotherapy, injury, adhesions and infection^23^, and can be modelled *ex vivo* by TGFβ1 treatment of peritoneal explants^49^,^50^. These features make explanted peritoneal tissue a relevant model to test the applicability of our interfacing material in a model of tissue fibrosis^21^. Towards that end, we explanted the abdominal wall of wild-type C57BL6N mice, interfaced laminin β1-fragment-functionalized pDA-coated PCL membranes directly with the mesothelium and incubated the tissues for 8, 24 and 120 h in the presence or absence of TGFβ1 (Fig. 6a). Explants were also incubated without membranes, with membranes functionalized with full-length laminin-111 and with non-functionalized pDA membranes to act as controls. Fresh tissues were processed for histological evaluation to observe the changes associated with fibrosis as a response to manipulations and incubation alone.

Explants incubated in the absence of TGFβ1 for 8, 24 or 120 h maintained a mostly intact mesothelial layer similar to that seen in freshly isolated peritoneal tissues. Treatment with TGFβ1 caused a clear thickening of the mesothelium (Supplementary Fig. 12). In the absence of TGFβ1, interfacing pDA-coated PCL membranes with and without immobilized laminin β1-fragment did not appear to have an impact on the histological appearance of the mesothelial layer (Supplementary Fig. 13). Importantly, the changes associated with TGFβ1 treatment were considerably mitigated in explanted tissues interfaced with membranes functionalized with the laminin β1-fragment, which maintained a mesothelial layer with integrity and E-cadherin staining comparable to that observed in tissues that were incubated in the absence of TGFβ1 (Fig. 6b). Further, α-SMA and fibronectin stainings were markedly weaker, suggesting that samples interfaced with the laminin β1-fragment comprised a decreased number of matrix-producing α-SMA-expressing fibroblasts compared to tissues interfaced with membranes without functionalization.

The observations from the histological appearance of the peritoneal tissues interfaced with functionalized electrospun membranes were further supported by a near-complete abrogation of the effect of TGFβ1 on active MMP2 released from the explants (Fig. 6c,d). Gelatin zymographs clearly indicate that active MMP2 levels of tissue explants interfaced with the laminin β1-fragment membranes were comparable to those of tissues that did not receive TGFβ1 treatment (Fig. 6d). These changes in active MMP2 levels were measured as early as 8 h after initiation of the TGFβ1 treatment and up to 120 h (Fig. 6c,d). This effect of the β1-fragment on active MMP2 levels could not be detected when tissue explants were interfaced with full-length laminin-111-coated membranes, further supporting the cryptic nature of the fragment activity (Supplementary Fig. 14). Taken together, these results strongly suggest regulatory potential for the cryptic laminin β1-fragment to actively counteract the consequences of TGFβ-induced MMP2 expression in peritoneal mesothelial cells.

In order to test the rescue potential of the fragment-functionalized membranes in a different tissue type, we isolated mouse bladders from B6.Cg-Tg(CAG-DsRed*MST)1Nagy/J, interfaced the urothelium with pDA-coated PCL membranes with and without immobilized laminin β1-fragment and incubated the tissues for 24 h in the presence or absence of TGFβ1, in the same approach as that used for peritoneal tissue explants. Gelatin zymographs of conditioned media demonstrated that active MMP2 levels in the conditioned media of tissue explants interfaced with the laminin β1-fragment membranes were comparable to those of tissues that did not receive TGFβ1 treatment, while tissue interfaced with non-functionalized membranes and treated with TGFβ1 showed the expected increase in active MMP2, similar to tissue samples that were not interfaced with pDA-coated PCL membranes (Supplementary Fig. 15). Further, in urothelium tissue interfaced with fragment-functionalized membranes, E-cadherin protein expression in the presence of TGFβ1 was rescued, while a decrease in E-cadherin protein levels was measured in tissue samples interfaced with non-functionalized membranes or no membranes (Supplementary Fig. 15). Continued research efforts will aim to enhance the clinical translatability of this novel approach by developing materials tailored specifically for different tissue types and surgical approaches.

### Implanted membrane reduces peritoneal fibrosis *in vivo*

**Figure 7:**
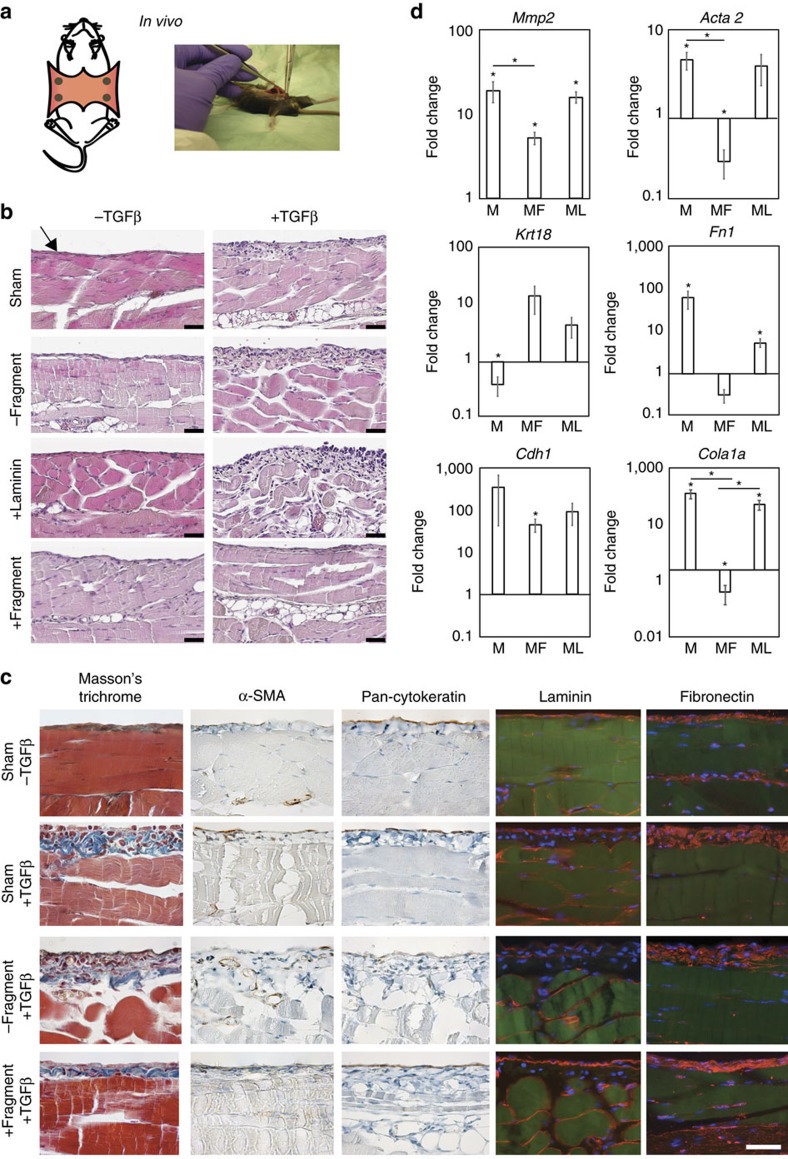
Membranes implanted in a mouse model of peritoneal fibrosis. (**a**) *In vivo* study: the abdomen was opened and four membranes were sutured to the peritoneum. After 7 days, empty or TGFβ1-adenoviral vectors were injected into the abdominal cavity and the mice were killed 8 days later. (**b**) Representative H&E images of peritoneal tissue of mice administered empty or TGFβ1-expressing adenoviral vectors for 8 days after implantation of membranes with (+fragment) or without (−fragment) laminin β1-fragment or full-length laminin-111 (+laminin). Sham refers to mice that underwent the full surgical procedure but did not have membranes implanted. Arrow pointing at the implantation site of the biomaterial on the mesothelium. Scale bars, 40 μm. (**c**) Corresponding representative immunohistochemical and immunofluorescent images of peritoneal tissue stained with Masson's Trichrome, α-SMA, pan-cytokeratin, laminin and fibronectin (antibody labelling, red; DAPI, blue; autofluorescence, green). Scale bars, 50 μm (applicable to all images). (**d**) Gene expression profile of peritoneal tissue extracts. M indicates non-functionalized membranes, MF and ML indicate membranes with immobilized laminin β1-fragment (MF) and full-length laminin (ML). *y* axis: log(2^(−ΔΔct)^). All data are normalized to GAPDH and to sham that received a TGFβ1-expressing adenoviral vector injection. *P<0.05.

To further demonstrate the potential of our material-based strategy to prevent fibrosis, we performed a proof-of-concept study in an established animal model of peritoneal tissue fibrosis^51^. We implanted pDA-coated PCL membranes with and without immobilized laminin β1-fragment or recombinant full-length laminin-111 into mice. Four membranes of the same composition were attached by a single suture on the peritoneal membrane of a mouse (Fig. 7a). Seven days after implantation, adenoviral vectors driving the expression of the active form of TGFβ1 (ref. 52) were administered intraperitoneally, while empty vectors were used as controls. Eight days after intraperitoneal virus injection (for a total implantation time of 15 days), mice without implanted material (sham) showed a significant fibrotic response to the TGFβ1 adenoviral treatment, demonstrated by thickening of the peritoneal tissue, deposition of collagen and fibronectin, the presence of α-SMA-positive myofibroblasts in the interstitium, a disrupted basement membrane, and a decrease in cytokeratin expression (Fig. 7b,c). Of note, peritoneal tissues that were previously interfaced with laminin β1-fragment-functionalized membranes exhibited less tissue thickening, less collagen and fibronectin deposition, less α-SMA-positive cells, a mesothelial cell layer expressing cytokeratin and an intact basement membrane in the area directly under the membrane. Conversely, peritoneal tissues interfaced with pDA-coated PCL membranes without fragment immobilization or functionalized with full-length laminin-111 showed a similar fibrotic morphology to tissues of sham models (Fig. 7b,c).

Gene expression data of tissue samples that were directly interfaced with the biomaterials during the experiment show an increase in *Mmp2, Acta2, Col1a1* and *Fn1*, as well as a decrease in *Krt18* expression in tissue samples that were interfaced with non-functionalized or laminin-111-functionalized pDA-coated PCL membranes relative to sham controls. However, the presence of the β1-fragment on the pDA-coated PCL membranes led to a significant decrease in *Mmp2, Col1a1* and *Acta*2 gene expression levels (Fig. 7d). It is interesting to note that the gene expression of *Cdh1* was elevated compared to sham controls in tissues under all three types of membranes. It should be mentioned that implanting the different pDA-coated PCL membranes in mice treated with empty adenoviral vectors also led to minor gene expression changes. *Cdh1, Col1a1* and *Krt18* were upregulated, while *Mmp2, Acta2* and *Fn1* showed modest upregulations (Supplementary Fig. 16). The effects of the material were restricted to the area interfaced with the membranes as tissue samples, taken away from the sites covered by the material did not exhibit an effect compared to sham at the gene expression level (Supplementary Fig. 17). Moreover, morphological changes due to the implanted materials were also constrained to the tissue area directly interfaced with the membranes (Supplementary Fig. 18). Evaluation of F4/80 labelling further showed that implantation of the pDA-coated PCL membranes did not increase the inflammatory response compared to sham treated with TGFβ1 (Supplementary Fig. 19).

Taken together, these results comprise a demonstration of the potential of our approach, whereby we interface the mesothelium of peritoneal tissues with a laminin β1-fragment-functionalized membrane to mitigate fibrosis in response to TGFβ1.

## Discussion

In this work, we developed a biomaterial-based strategy to inhibit tissue fibrosis using a biomimetic approach, whereby we interface epithelial (or mesothelial) cells with high concentrations of a cryptic fragment of the laminin β1-chain exposed by the action of MMP2, to elicit a specific inhibition of MMP2 activity, the gene and protein expression of EMT-related molecules and morphological changes associated with fibrosis. We have demonstrated the potential of this novel strategy to target tissue fibrosis *in vitro*, as well as in explanted mouse peritoneal membrane tissues, explanted mouse bladder urothelium tissue and in an *in vivo* model of peritoneal fibrosis. Other groups have previously reported on the development of materials that promote EMT^53^,^54^. As the electrospun membrane design harnesses the specific biological activity of a cryptic ECM fragment that acts via interactions with α3β1-integrin upon interfacing with epithelial/mesothelial tissue, it is likely to circumvent off-target secondary effects that have plagued previous therapeutic approaches aimed at addressing fibrosis. This strategy is particularly suited to interventions in which the site of fibrogenesis is localized, known or can be predicted with a high degree of accuracy. As such, the approach could be adapted to minimize the risks of peritoneal adhesions that can result from abdominal surgeries, or combined with peritoneal dialysis catheters to reduce local effects of the implanted material. It is worth noting that this approach requires interfacing with the apical side of the peritoneal mesothelium.

The significance of the contribution of EMT to tissue fibrosis is still under debate in certain fibrotic tissue types. However, the degradation of the basement membrane by MMPs, specifically MMP2, is an inevitable pre-requisite to enable cell migration and the disintegration of epithelial tissue. In this work, we show that a degradation product of the basement membrane protein laminin can present cryptic signals that alter EMT-related signalling pathways and decrease MMP2 expression and activity in NMuMG cells and primary mouse peritoneal mesothelial cells treated with TGFβ1. The proposed mechanism, possibly downstream of α3β1-integrin, involves a change in expression and nuclear translocation of p-smad3 and snail. Moreover, interaction with the laminin β1-fragment alters MT1-MMP expression, a known activator of MMP2. Detailed molecular mechanisms need to be further elucidated in the future; however, our data clearly demonstrate that this signalling mechanism can be harnessed to decrease MMP2 activity in NMuMG cells treated with TGFβ1 *in vitro*. These results, as well as the demonstration that the laminin β1-fragment-functionalized membrane can abrogate the release of active MMP2 from explanted peritoneal mesothelium and explanted bladder urothelium treated with TGFβ1, are extremely encouraging in light of the recognized importance of MMP2 as a potential target for the treatment of tissue fibrosis. Further *in vivo* demonstration that our functionalized membrane decreases the fibrotic response of the peritoneal membrane to TGFβ1 treatment in mice highlights the potential of this novel therapeutic strategy. Owing to the fact that EMT/MMT is only one of a number of sources of the myofibroblasts accumulating in fibrotic tissues, it is unlikely that the effect of the laminin fragment-functionalized membrane observed here is solely linked to the inhibition of MMT in resident mesothelial cells.

In conclusion, we demonstrate the potential of basement membrane fragments that can be incorporated into synthetic polymer matrices to efficiently modulate MMP expression and activity. In a broader context, this work will hopefully encourage the characterization and application of the untapped reservoir of biological activities hidden within the ECM that can be harnessed for biomaterial functionalization beyond supporting only cell adhesion. While we have established the potential of the material developed in this work for rescuing peritoneal tissue from the effects of TGFβ1, we are confident that the fragment presentation strategy can be tailored to address the modulation of MMP expression in a range of tissues, given the broad range of tissue types in which the laminin β1-chain is found.

## Methods

### Recombinant laminin fragment production

Recombinant laminin-111 fragment β1-LnLe1-4 was produced as described previously^29^,^55^. Human embryonic kidney HEK293 c18 cells (American Type Culture Collection) were cultured at 37 °C with 5% CO_2_ in DMEM/nutrient mixture F-12 (Invitrogen) containing 10% (v/v) fetal bovine serum (FBS; Life Technologies), 2 mM glutamine (Gibco), 10 units per ml penicillin (Gibco), 100 μg ml^−1^ streptomycin (Gibco) and 250 μg ml^−1^ Geneticin (Gibco), transfected with the expression vector using FuGENE 6 (Roche Diagnostics) and selected with 1 μg ml^−1^ puromycin (Sigma). Transfected cells were grown to confluence in HYPERFlask vessels (Corning), washed twice with PBS and incubated with serum-free medium for 3 weeks with weekly medium changes. The laminin β1-fragment was purified from serum-free conditioned medium by nickel affinity chromatography using 5 ml HisTrap FF columns (GE Healthcare) and an ÄKTA FPLC System (GE Healthcare). The purified protein was concentrated to 1 mg ml^−1^ using Amicon ultracentrifugal 10 K molecular weight cutoff (MWCO) units (Fisher Scientific UK), analysed by SDS–PAGE and stored at −20 °C.

### Scanning electron microscopy

The surface morphology of the pDA-coated PCL membranes was imaged using a JEOL JSM 5610 LV SEM. Membranes were sputter-coated with ∼40 nm Au (Emitech K550) before imaging. The samples were observed under secondary electron imaging at an accelerating voltage of 5 kV and a working distance of 15 mm. The average fibre diameter was determined using ImageJ by measuring at least 120 fibres from six micrographs taken from three samples per condition.

### Membrane fabrication

PCL (average Mn 80,000 Da; Sigma) was dissolved in 1,1,1,3,3,3-hexafluoroisopropanol (Sigma) overnight to obtain a 12% (w/v) solution. The PCL solution was electrospun through a 18-gauge blunt-tip needle positioned 10 cm from a voltage-driven rotating mandrel (width=6.3 cm; diameter=10.0 cm) adjusted to a linear velocity of 1 m s^−1^. A programmable syringe pump (Kd Scientific; Model KDS 100 CE) was used to control the flow rate at 2 ml h^−1^. A voltage of 16 kV was applied to the needle with a high-voltage power supply (Glassman High Voltage Inc.; Series WR), while the rotating mandrel was grounded. A total of 5 ml of PCL solution was electrospun to produce a fibrous membrane ∼200 μm in thickness with most fibres in the 0.5–2.0 μm diameter range.

### Preparation of a pDA coating on PCL membranes

Electrospun PCL membranes were cut into 6 or 8 mm diameter discs and plasma-treated (Gala Instrumente; Plasmaprep 5) in air at a pressure of 0.4 mbar for 30 s on each side to render them hydrophilic and ensure a uniform application of the coating^56^. The membranes were then immersed in a 2 mg ml^−1^ dopamine (Sigma) solution dissolved in 10 mM Tris (Sigma; pH 8.5) under constant mixing. Samples were incubated for 1, 4 or 24 h, and 4 h incubation was deemed optimal and used for all cell culture experiments. All samples were washed thoroughly in distilled H_2_O overnight with the H_2_O changed three times, and dried before further testing.

### Quantification of BSA immobilization

pDA-coated membranes were sterilized in 70% (v/v) ethanol, washed three times in PBS and incubated at 37 °C in 200 μl of a 25 μg ml^−1^ BSA (Sigma) solution in PBS (Gibco) for 24 h. After the incubation period, the supernatant was removed and the protein content was measured using a fluorescamine assay (Sigma). Briefly, 75 μl aliquots of the supernatants were incubated with 25 μl of 100 μg ml^−1^ fluorescamine in acetone and the fluorescence intensity was measured at an excitation of 385 nm and an emission of 465 nm. A standard curve with a concentration range of 1–50 μg ml^−1^ was prepared with BSA in PBS. PCL membranes after plasma treatment but without pDA coating were used as controls. For each condition, membranes incubated in PBS only were also tested as blanks. The blank values were subtracted from the protein concentration measured in the supernatant. The amount of immobilized BSA was determined indirectly by calculating the difference in the protein content measured in solutions incubated without membranes and that in wells containing the membranes. Immobilized BSA was normalized to the membrane weight. The experiment was repeated with the laminin β1-fragment. The amount of laminin β1-fragment immobilized on membranes was measured in the same way as described for BSA and expressed as a percentage of the amount of fragment measured in the solutions that were not incubated with membranes.

### Visualization of laminin fragment on membranes

Recombinant laminin fragment (100 μg) was dissolved in ammonium bicarbonate (0.1 M, pH 9.0) and FITC isomer I (50 μg, Sigma) previously dissolved in dimethylsulphoxide was added. The reaction mixture was allowed to react overnight at 4 °C and dialysed (3,500 Da MWCO) against water overnight. Sterilized pDA-coated PCL membranes were incubated at 37 °C in 200 μl of 10 or 100 μg ml^−1^ FITC-labelled laminin β1-fragment in PBS for 8 h based on data, suggesting a nonsignificant difference in the amount of BSA immobilized on pDA-coated PCL membranes after 8 or 24 h incubations (data not shown). After the incubation period, membranes were washed three times in PBS. For total internal reflection fluorescence microscopy, samples were placed on glass substrates (glass bottom dish, MatTek) in PBS and imaged using a Zeiss Axiovert 200 manual inverted microscope with a 488 laser diode, a × 100/1.45 W alpha Plan-Fluar objective and back illuminated EM-CCD camera (Hamamatsu C9100-13). For confocal imaging, a laser-scanning confocal fluorescence microscope (SP5, Leica) with a HC PL APO 10 × 0.40 CS air objective was used. On the basis of these data and previous work^16^, 10 μg ml^−1^ recombinant laminin β1-fragment was used as a coating concentration for all cell culture experiments.

### Raman spectroscopy

The molecular composition of the pDA-coated PCL membranes was analysed by Raman spectroscopy. Spectra from the dried samples were measured using the WITec Alpha 300R+ Confocal Raman Microscope System with a WITec 532 nm excitation laser and a 50 μm optical fibre. Spectra of control PCL membranes were measured with a Zeiss EC Epiplan × 50/0.75 high definition (HD) objective under 30.6 mW laser power, with 0.5 s integration time and 10 accumulations. Spectra of the pDA-coated PCL membranes were measured with a Zeiss EC Epiplan-Neofluar × 10/0.25 HD differential interference contrast objective under 40.4 mW laser power, 1 s integration time and 10 accumulations. Three membranes were measured for each condition and spectra were obtained from three different points on each membrane.

### Coating of membranes with laminins

pDA-coated PCL membranes were sterilized in 70% (v/v) ethanol, washed three times in PBS and incubated at 37 °C in 200 μl of 10 μg ml^−1^ laminin β1-fragment in PBS, recombinant mouse full-length laminin-111 (a kind gift of Sergey Rodin) or human recombinant laminin-521 (Biolamina) in PBS for 8 h. After the incubation period, membranes were washed three times in PBS.

### Cell culture

NMuMG cells (CRL-1636; American Type Culture Collection) were maintained in DMEM (Life Technologies) with 10% (v/v) FBS (Life Technologies), 10 μg ml^−1^ insulin (Sigma) and 100 U ml^−1^ penicillin–streptomycin (Life Technologies) at 37 °C with 5% CO_2_. Cells were collected with 0.05% (v/v) 1 × trypsin-EDTA (Life Technologies) and passaged at 80–90% confluence. To facilitate cell seeding on pDA-coated PCL membranes, cell chambers were prepared from domed 8-cap and tube strips for PCR (Axygen). Using a razor blade, the domed top of the caps was cut off and the remaining frames were separated from each other. The conical portion of the tubes was also cut off to yield an open rim. The frames, rims and pDA-coated PCL membranes were sterilized in 70% (v/v) ethanol for 30 min and rinsed three times with PBS. Each membrane was then sandwiched between a cap and a rim under sterile conditions, and the assembled cell chambers were incubated in 1% (w/v) BSA (Sigma) in PBS overnight at 37 °C. Membranes that had not previously been incubated with the laminin β1-fragment, recombinant full-length laminin-111 or laminin-521 were also incubated in BSA at this stage to serve as controls. Cells were harvested as described above and seeded at a density of 10,000 cells per membrane. The EMT was initiated both on tissue culture plastic and on materials in selected samples by complementing the medium with 10 ng ml^−1^ TGFβ1 (Sigma) once cells reached at least 70% confluence. Control experiments were also carried out with plasma-treated PCL membranes without pDA coating and on tissue culture plastic. Cells were regularly tested for mycoplasma contamination.

Primary mouse peritoneal mesothelial cells were isolated from female, 11-week-old B6.Cg-Tg(CAG-DsRed*MST)1Nagy/J mice. All procedures were performed according to ethical permits issued by the local Ethical Committee at Karolinska Institutet, Stockholm (N240/15, Juha Ojala). The mice were housed with regulated light and dark cycles under pathogen-free conditions at the Scheele Animal Facility (Karolinska Institutet) with access to food and water *ad libitum*. The mice were killed by isoflurane overdose followed by cervical dislocation or overdose of isoflurane anaesthesia, shaven and sterilized with ethanol. Skin was removed and the peritoneal wall was collected. Peritoneal tissue was submerged in 4 ml 0.05% (v/v) 1 × trypsin-EDTA (Life Technologies) and incubated at 37 °C for 15 min with regular mixing. Four millilitres of DMEM-F-12 (Life Technologies) with 20% (v/v) FBS (Life Technologies), 100 U ml^−1^ penicillin–streptomycin (Life Technologies) and 2% (v/v) Biogro-2 (Biological Industries Israel Beit-Haemek Ltd.) were then added and samples were centrifuged for 5 min at 300 r.c.f. The remaining tissue was removed and cells were plated on collagen-coated tissue culture plastic (Thermo Fisher Scientific). Cells were trypsinized and used for experiments 24 h after isolation.

### Isolation and culture of mouse peritoneal wall *ex vivo*

All animal experiments were approved by the local ethical committee at the North Stockholm district court (N240/15, Juha Ojala). The mice were housed with regulated light and dark cycles under pathogen-free conditions at the Scheele Animal Facility (Karolinska Institutet) with access to food and water *ad libitum*. For the isolation of peritoneal wall samples, 6-week-old C57BL6N male mice were killed with cervical dislocation, shaved and carefully wiped with 70% (v/v) ethanol (Sigma) to avoid contamination. The skin layer was removed and the peritoneal wall was excised and placed on a sterile tissue culture plate as one piece with the mesothelial layer facing up. While keeping the surface moist with DPBS (Gibco, Life Technologies) the sample was cut under sterile conditions into ∼0.5 × 0.5 cm^2^ pieces. Some of the pieces were used as untreated controls and immediately snap-frozen in optimal cutting temperature compound (OCT) (Sakura). Remaining samples were interfaced on the apical side with pDA-coated PCL membranes without immobilized laminin β1-fragment, with immobilized laminin β1-fragment, with full-length laminin-111, or cultured without interfaced membranes, placed in 24-well plates coated with polydimethylsiloxane (Dow Corning; SYLGARD 184) and submerged with 250 μl 50% (v/v) Opti-MEM, 25% (v/v) FBS, 24% (v/v) Hank's balanced salt solution (HBSS) and 100 U ml^−1^ penicillin–streptomycin (Life Technologies) at 37 °C with 5% CO_2_ with and without treatment with 1 or 10 ng ml^−1^ TGFβ1 (Sigma) for 8, 24 or 120 h.

### Isolation and culture of mouse bladder *ex vivo*

Female 11-week-old B6.Cg-Tg(CAG-DsRed*MST)1Nagy/J mice were used for the isolation of urinary bladder urothelium. All procedures were performed according to ethical permits issued by the local Ethical Committee at Karolinska Institutet, Stockholm (N240/15, Juha Ojala). The mice were killed by isoflurane overdose, followed by cervical dislocation or overdose of isoflurane anaesthesia, shaven and sterilized with ethanol. Skin was removed, and the peritoneal wall was collected and processed for primary cell isolation (see Cell culture subsection). Next, the bladder was inflated with 1 ml of sterile HBSS (Life Technologies) and ligated to keep the bladder wall extended after the bladder was removed. The bladder wall was incised and the two halves were extended so that the inner urothelial lining was facing up. pDA-coated PCL membranes with or without immobilized laminin β1-fragment were stapled on the apical side of the urothelial cell layer on top of a polydimethylsiloxane-coated dish (Dow Corning; SYLGARD 184) in order to constrain them during *ex vivo* culture. Tissue samples were used as fresh tissue controls and controls incubated without the pDA-coated PCL membranes. Samples were submerged with 200 μl 50% (v/v) MCDB153 (Biological Industries Israel Beit-Haemek Ltd.), 50% (v/v) DMEM Life Technologies, 0.1 mM Ethanolamine, 0.1 mM Phosphoethanolamine, 15 μg ml^−1^ adenine, 0.5 μg ml^−1^ hydrocortisone, 5 μg ml^−1^ insulin (all Sigma-Aldrich) and 100 U ml^−1^ penicillin–streptomycin (Life Technologies) at 37 °C with 5% CO_2_ with and without treatment with 10 ng ml^−1^ TGFβ1 (Sigma) for 24 h.

### Adenovirus production

HEK 293A cells were infected with a control adenovirus or with an adenovirus encoding active TGFβ1, kindly provided by Fernando Rodríguez-Pascual^57^. Four days post infection, adenoviral particles were recollected and purified with the Adeno-X Maxi Purification Kit (Clonetech Laboratories, Mountain View, CA, USA) and later titrated with the Adeno-X Rapid Titer Kit (Clonetech).

### *In vivo* animal studies

C57BL6J 5-week-old mice were used (Charles River Laboratories, Barcelona, Spain). Food and water were provided *ad libitum* to the animals. The experimental protocols followed were in accordance with the National Institutes of Health Guide for Care and Use of Laboratory Animals and were approved by the Animal Ethics Committee of the ‘Unidad de Experimentación Animal' del Centro de Biología Molecular ‘Severo Ochoa' (Manuel López-Cabrera. Madrid, Spain). The mice were put to sleep with 4% isoflurane inhalation and kept asleep during the operation on a heated pad under 2% isoflurane inhalation. An anterior midline incision was made through the abdominal wall and peritoneum using a pair of scissors. Two membranes of the same type (membrane alone, membrane immobilized with laminin β1-fragment or membrane immobilized with full-length laminin-111) were attached to the apical side of the peritoneum on each side of the incision using 6-0 silk sutures and spaced ∼1 cm apart. Seven days post-membrane implantation, animals were intraperitoneally inoculated with 1 × 10^8^ infection-forming units of control or TGFβ1-encoding adenovirus. Animals were killed at 15 days post implantation and 8 days post transfection. The peritoneum and overlying muscle surrounding each scaffold were resected with an equal area of control tissue.

For PCR analysis, the left and right superior samples were subdivided into tissue directly interfaced with the membrane and control tissue, taking care to discard the midline incision site and the margin around the implant. For control and sham animals, an equal area of control tissue was obtained from the same anatomical locations. The membranes were detached from the underlying peritoneum before tissue samples were stored in RNAlater (Ambion) at room temperature for no more than 7 days before homogenization.

For histological analysis, the left and right inferior tissue samples were bisected through the midline, retaining both peritoneum in contact with the implant and control tissue in each. The two halves were processed for paraffin sectioning and cryosectioning. Samples for paraffin-embedding were fixed in 4% (v/v) buffered formalin overnight at room temperature. Samples for cryosectioning were snap-frozen in OCT compound.

### Metabolic activity assay

An alamarBlue assay (Life Technologies) was performed before EMT initiation (1 day after seeding) and 24 h after EMT initiation according to the manufacturer's instructions. Briefly, constructs were incubated in 750 μl of basic growth medium defined above and supplemented with 8% (v/v) alamarBlue solution for 2 h at 37 °C. Empty wells and membranes without cells were also incubated as blanks. The fluorescence of resulting aliquots was measured at an excitation of 540 nm and an emission of 580 nm. The blank fluorescence was subtracted from the fluorescence measured in membranes seeded with cells.

### Gene expression

RNA of NMuMG cells was isolated using an RNeasy Mini Kit (Qiagen) according to the manufacturer's instructions. RNA of peritoneal tissue samples was extracted using Trizol/Chloroform (Invitrogen, Sigma-Aldrich) and lysing kits (Precellys) as described previously^58^. QuantiTect Reverse Transcription Kit (Qiagen) and QuantiTect SYBR Green PCR Kit (Qiagen) were used to perform reverse transcription and qPCR. Thermocycling and SYBR Green detection were performed on a Corbett Rotorgene 6000 (Qiagen) with extension at 72 °C and denaturing at 95 °C. All primers were annealed at 55 °C. Data were analysed using the Pfaffl method. The following primers were used: *Mmp2* (For 5′-ATGGCAAGTATGGCTTCTG-3′ and Rev 5′-GTAGGAGGTGCCCTGGAAG-3′), *Gapdh* (For 5′-TGGTATCGTGGAAGGACTCATGA-3′ and Rev 5′-ATGCCAGTGAGCTTCCCGTTCAG-3′), *Mmp9* (For 5′-TGTACCGCTATGGTTACAC-3′ and Rev 5′-CCGCGACACCAAACTGGAT-3′), *Cdh2* (For 5′-AGGGTGGACGTCATTGTAGC-3′ and Rev 5′-CTGTTGGGGTCTGTCAGGAT-3′), *Cdh1* (For 5′-CGAGAGAGTTACCCTACATA-3′ and Rev 5′-GTGTTGGGGGCATCATCATC-3′), *Snai2* (slug; For 5′-CATCCTTGGGGCGTGTAAGT-3′ and Rev 5′-ATGGCATGGGGGTCTGAAAG-3′), *Snai1* (snail; For 5′-CACACGCTGCCTTGTGTCT-3′ and Rev 5′-GGTCAGCAAAAGCACGGTT-3′), *Col1A1* (For 5′-TAAGGGTCCCCAATGGTGAGA-3′ and Rev 5′-GGGTCCCTCGACTCCTACAT-3′), *Fn1* (For 5′-CCCTATCTCTGATACCGTTGTCC-3′ and Rev 5′-TGCCGCAACTACTGTGATTCGG-3′), *Acta2* (For 5′-CCTGACTGAGCGTGGCTATT-3′ and Rev 5′-CATAGCACAGCTTCTCCTTGA-3′), *Krt18* (For 5′-ACCCTCCAGACCTTGGAGAT-3′ and Rev 5′-TCCATCTGTGCCTTGTATCG-3′), *Tjp1* (For 5′-CCATGGCCTCAAGTTCCTG-3′ and Rev 5′-GGCTCCAACAAGGTAATTCG-3′)^29^.

### Antibodies

Antibodies for immunostaining of NMuMG and mouse mesothelial cells are as follows: anti-E-cadherin (1:250, Cell Signaling Technology), anti-αSMA (1:750, Sigma), anti-ZO-1 (1:400, Abcam), anti-N-cadherin (1:200, Santa Cruz Biotechnologies), anti-S100A4 (1:250, Dako), goat anti-mouse Alexa fluor-647 (1:500, Life Technologies), goat anti-rabbit Alexa fluor-488 (1:500, Life Technologies), donkey anti-mouse Alexa fluor-488 (1:750, Life Technologies) and donkey anti-rabbit Alexa fluor-488 (1:750, Life Technologies). F-actin was stained with Alexa Fluor-488 Phalloidin (1:1,000, Life Technologies), and the nuclei were labelled with 300 nM 4′,6-Diamidino-2-phenylindole dihydrochloride (DAPI, Sigma-Aldrich). For immunostaining of tissue sections, antibodies used were as follows: anti-E-cadherin (1:250, Abcam), anti-α-SMA (1:3,000, Sigma-Aldrich), anti-fibronectin (1:200, Abcam), anti-cytokeratin (1:500, Sigma-Aldrich), anti-laminin (1:200, Abcam), anti-F4/80 (1:50, Abcam), goat anti-rabbit Alexa fluor-647 (1:500, Life Technologies), goat anti-rat Alexa fluor-647 (1:500, Life Technologies) and goat anti-mouse Alexa fluor-488 (1:1,000, Life Technologies). For immunprecipitation, antibodies used were as follows: anti-α3-integrin (A1920 Millipore), anti-mouse IgG (Milipore) and anti-β1-laminin fragment (custom-made in rabbit). For immunoblotting, antibodies used were as follows: anti-snail (C15D3, Cell Signaling), anti-MT1-MMP (D1E4, Cell Signaling), anti-phospho-smad3 (C25A9, Cell Signaling), anti-smad3 (C67H9, Cell Signaling), anti-phospho-smad2 (Ser245/250/255, Cell Signaling), anti-smad2 (D43B4, Cell Signaling), anti-GAPDH (D16H11, Cell Signaling), anti-pY654-β-catenin (Tyr-654, ECM Biosciences), anti-laminA/C (Ser22, Cell Signaling), anti-slug (C19G7, Cell Signaling), anti-E-cadherin (24E10, Cell Signaling), anti-α-SMA (Cell Signaling), anti-β-actin (ACTBD11B7, Santa Cruz Biotechnology), goat anti-Rabbit IgG (H+L) secondary antibody, goat anti-Mouse IgG (H+L) secondary antibody and horse radish peroxidase conjugate (Thermo Scientific).

### Immunofluorescence of NMuMG and mesothelial cells

Cells were washed with PBS (Life Technologies), NMuMG cells were fixed in 3.7% (v/v) formaldehyde and mesothelial cells in 4% (v/v) EM-grade paraformaldehyde (Sigma) for 15 min at room temperature and washed again. Mesothelial cell samples and selected NMuMG cell samples for phalloidin, α-SMA, FSP-1 and N-cadherin labelling were permeabilized in 0.25% (v/v) Triton X-100 (Sigma) in PBS for 5 min and washed three times. Samples were then blocked in 3% (w/v) BSA (phalloidin immunolabelling (Sigma), 10% (v/v) donkey serum (all other antibodies) or 5% (v/v) donkey serum (mesothelial cell samples) in PBS 1 h at room temperature. Samples were incubated in primary antibodies in blocking solution for 40 min at room temperature (phalloidin immunolabelling), or overnight (all other antibodies) at 4 °C, washed four times in PBS (30 s and 1, 5 and 15 min) and incubated in secondary antibodies in blocking solution (containing DAPI) for 1 h at room temperature. Samples were washed again four times in PBS (30 s and 1, 5 and 15 min). The samples were mounted on glass coverslips with a drop of VECTASHIELD mounting medium (Vector Laboratories, UK) to help prevent photobleaching during imaging. Images were taken on a Leica SP5 inverted confocal microscope, using a HCX PL APO CS × 63 (1.40) immersion oil objective or on a Zeiss LSM 800 confocal microscope with Z stacks taken with 0.89 μm step size. Actin fibre orientation was evaluated using the OrientationJ plug-in for ImageJ^59^.

### Histology and immunohistochemical analysis of animal tissues

For explants, tissue samples were snap-frozen in OCT (Sakura). Cryosections (5–15 μm) were either air-dried and stained with haematoxylin and eosin using a Tissue stainer TST 33 or fixed in 100% (v/v) cold acetone (Sigma) for 20 min, blocked with 5% (v/v) goat serum (Life Technologies) for 1 h at room temperature and incubated in primary antibody in 5% (v/v) goat serum for 1 h at 37 °C, washed in PBS and then stained with secondary antibody and DAPI dihydrochloride (300 nM, Sigma-Aldrich) in 5% (v/v) goat serum for 45 min at 37 °C. Samples were mounted on SuperFrost Plus slides (Ted Pella) in the Fluoroshield mounting medium (Sigma), and images were taken with a Leica DM LB with Leica DFC 320 camera or a Leica DM RB with Retiga Exi camera (Q Imaging). For *in vivo* tissue samples, paraffin sections (5 μm) were cut and stained with haematoxylin and eosin and Masson's trichrome (DiaPath) to evaluate fibrosis. Immunohistochemical analysis was performed to visualize mesothelial cells (pan-cytokeratin) and pathologic fibroblasts (α-SMA; Sigma) using Real Target Retrieval Solution (DAKO) antigen retrieval and Mouse-Over-Mouse Polymer IHC Kit (Abcam), and counterstained with Harris Haematoxylin (Leica). Paraffin sections (5 μm) for immunofluorescent imaging of fibronectin (Abcam), laminin (Abcam) and F4/80 (Abcam) were treated with citrate antigen retrieval buffer (pH 6.0) at 60 °C overnight and blocked with 5% (v/v) goat serum (Life Technologies) in PBST for 1 h at room temperature. Primary antibodies were incubated overnight at 4 °C in blocking buffer, washed in PBST and then stained with secondary antibody and DAPI (Sigma-Aldrich) in blocking buffer for 1 h at room temperature. Samples were mounted with Fluoroshield mounting medium (Sigma) and images were acquired on an AxioImager M2 (Zeiss) with an Axiocam 503 mono camera (Zeiss).

### Immunoprecipitation

NMuMG cells were incubated with indicated antibodies for 1 h at 4 °C, washed with PBS (Gibco) and lysed using 50 mM Tris-HCl, 150 mM NaCl, 1% (v/v) Triton X-100, 0.1% (w/v) Na-deoxycholate and 1 mM EDTA (all Sigma-Aldrich), pH 7.5. Lysed cells were then sonicated for 55 s at 20% amplitude using a VibraCell sonicator, and passed through a 26-g needle three times. Cell lysated were centrifuged for 10 min and protein concentrations of supernatants were measured using a Bradford assay (Thermo Scientific). Protein-G sepharose beads (30 μl, Sigma-Aldrich) were incubated with 1 mg protein for 2 h at 4 °C. Proteins were eluted in Laemmli sample buffer by heating for 7 min at 100 °C and were analysed using immunoblotting with indicated antibodies.

### Immunoblotting

Cells and tissue samples were lysed in RIPA buffer with protease and phosphatase inhibitors (Santa Cruz Biotechnology). Lysates were incubated at 4 °C at 1,000 r.p.m. for 30 min and then centrifuged for 30 min at 16,000*g* at 4 °C. Supernatants were separated on SDS–PAGE and analysed using immunoblotting. Densitometric analysis was performed using ImageJ.

### Gelatin zymography

Active MMP2 in conditioned media was determined with gelatin zymography. For *in vitro* studies, 1 ml of conditioned medium was collected from each sample, filtered and concentrated by a factor of 10 using Amicon ultra-2 centrifugal filter units with ultracel-10 membranes (Merck Millipore). Protein concentration was measured using a NanoDrop spectrophotometer (Thermo Scientific) against medium incubated on membranes without cells (blanks). For *ex vivo* studies, 200 μl of conditioned media were collected, filtered as described above and used immediately. Ten per cent (w/v) gelatin gels were prepared (using gelatin from porcine skin, type A (Sigma)) or commercially available ten per cent (w/v) gelatin gels were used (Invitrogen). Samples were mixed with zymogram sample buffer (Bio-Rad) and proteins were resolved by SDS–PAGE. Gels were then washed in PBS with 2.5% (v/v) Triton X-100 for 1 h at room temperature and incubated in assay buffer (0.5 M Tris-HCl, pH 7.8, 2 M NaCl, 0.05 M CaCl_2_ and 0.2% Brij 35) overnight at 37 °C. Gels were then stained with 0.05% (w/v) Coomassie Blue G-250, 5% (v/v) methanol and 10% (v/v) acetic acid for 1 h at room temperature and subsequently de-stained in 5% (v/v) methanol and 10% (v/v) acetic acid. Gelatin gels were either scanned using the Licor Odyssey system (Licor) or with a ChemiImager 5500. Active MMP2 (Sigma) was used as a positive control. Densitometric analysis was performed using ImageJ.

### Collagen assay

NMuMG cells on PCL membranes were pooled (six per condition) and digested with papain (100 μg ml^−1^; Sigma) in a digestion buffer (5 mM ethylenediaminetetraacetic acid and 5 mM L-cysteine) for 24 h at 56 °C. The DNA content of papain digests was quantified using a Quant-IT PicoGreen kit (Invitrogen) as per the manufacturer's protocol. Aliquots of the papain digest were hydrolysed in 6 N HCl at 110 °C for 18 h. The resulting hydroxyproline content was determined by measuring the colour change spectrophotometrically at 560 nm following incubations with choramine-T and Ehrlich's reagent. The standard curve was generated with l-hydroxyproline (Sigma). The hydroxyproline content was used as an estimation of the collagen deposited by NMuMG cells on the PCL membranes and was normalized to DNA.

### Statistical analysis

Results are presented as mean±s.e.m. unless specified otherwise. Statistical analysis was performed using SPSS 21. Significance between two groups was determined by *t*-test. For protein binding, three independent experiments were performed with four replicates per condition in each experiment and results were analysed by analysis of variance with a Tukey *post hoc* test. For qPCR data, at least three independent experiments were performed with at least three replicates per condition in each experiment and samples were tested for normal distribution using Komogorov–Smirnov and Shapiro–Wilk tests, and homoscedasticity was verified using Levene's test. Differences were tested by *t*-test for independent samples. Immunofluorescence and zymography were performed on samples from at least three independent experiments. *Ex vivo* and primary mouse mesothelial cell experiments were performed with tissues/cells extracted from three mice with two replicates per animal for each condition. For the *in vivo* study, samples from five animals for each of the four conditions treated with TGFβ1-encoding adenovirus and three animals for each of the four conditions treated with empty adenovirus were obtained (including three controls—a total of 35 animals). Two replicates were obtained per animal from tissues directly under the membrane and two more from tissues away from the membrane. Histology was performed on samples from five animals per condition for haematoxylin and eosin and three animals per condition for Masson's Trichrome and immunohistochemistry. Empty and TGFβ1-encoding adenovirus was administered in a randomized way cage-wise. Differences were considered statistically significant when *P*<0.05 (*) and very significant when *P*<0.005 (**).

### Data availability

Supporting raw data is available upon request from m.stevens@imperial.ac.uk.

## Additional information

**How to cite this article:** Horejs, C.-M. *et al*. Preventing tissue fibrosis by local biomaterials interfacing of specific cryptic extracellular matrix information. *Nat. Commun.* **8**, 15509 doi: 10.1038/ncomms15509 (2017).

**Publisher's note:** Springer Nature remains neutral with regard to jurisdictional claims in published maps and institutional affiliations.

## Supplementary Material

Supplementary Information Supplementary Figures.
