# Supplementary material for: Preventing tissue fibrosis by local biomaterials interfacing of specific cryptic extracellular matrix information
Source: Nat Commun. 2017 Jun 8;8:15509. doi: 10.1038/ncomms15509 (PMC5472175; doi:10.1038/ncomms15509)
Supplement: Supplementary Information — Supplementary Figures. [file ncomms15509-s1.pdf]

Supplementary Figures

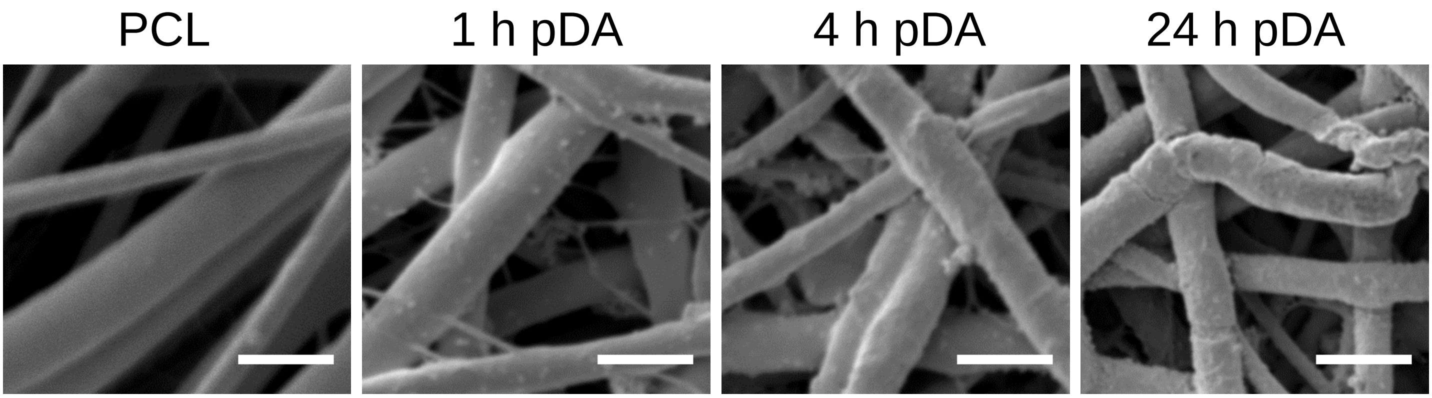

Supplementary Figure 1. Membrane characterization Scanning electron micrographs of air plasma-treated polydopamine poly(ε-caprolactone) (PCL) membranes prior to (PCL) and after 1 h, 4 h and 24 h incubation in a 2 mg ml^{-1} dopamine solution to apply a polydopamine (pDA) coating. Scale bars = 1 μm.

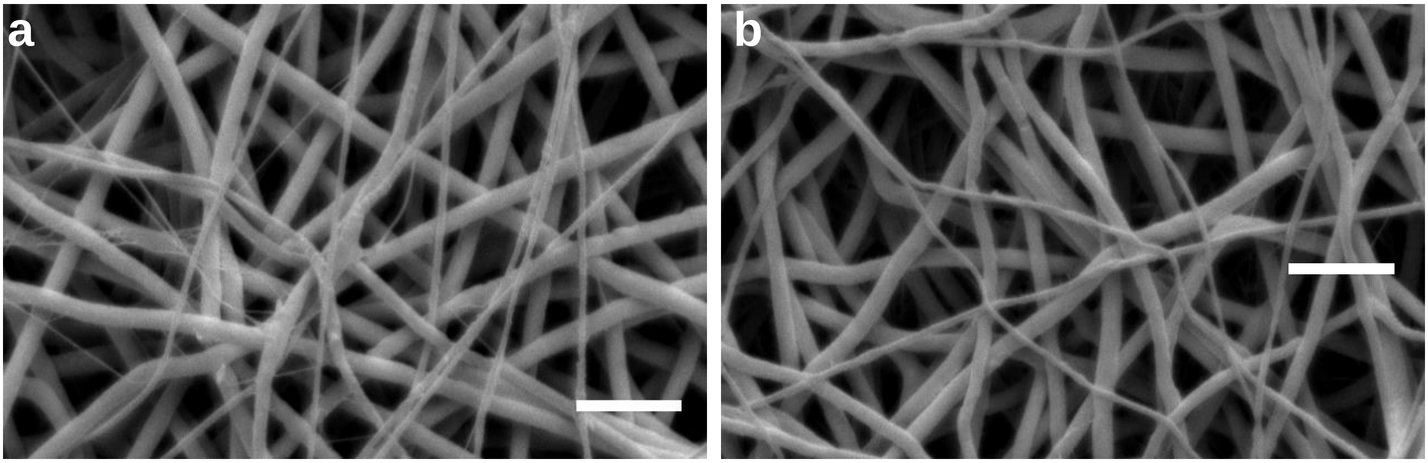

Supplementary Figure 2. Membrane characterization Scanning electron micrographs of polydopamine (pDA)-coated poly(ε-caprolactone) (PCL) membranes **a,** prior to and **b,** after plasma treatment. Scale bars = 10 μm; magnification: x2000.

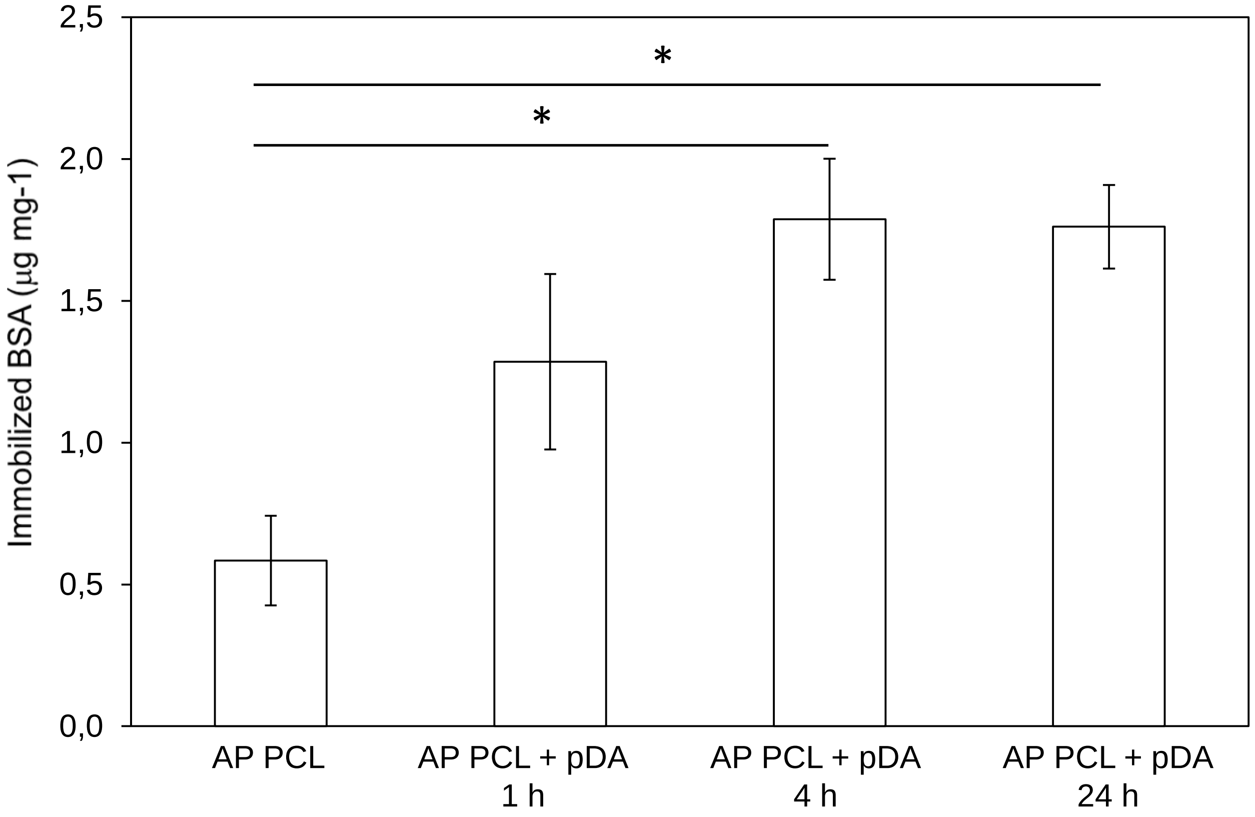

Supplementary Figure 3. Protein functionalization of membrane BSA binding on plasma-treated (AP) poly(ε-caprolactone) (PCL) membranes as a function of incubation time in a 2 mg/ml dopamine solution to apply a polydopamine (pDA) coating. BSA binding is normalized to the membrane weight. Data shown as mean ± standard deviation. * $p < 0.05$.

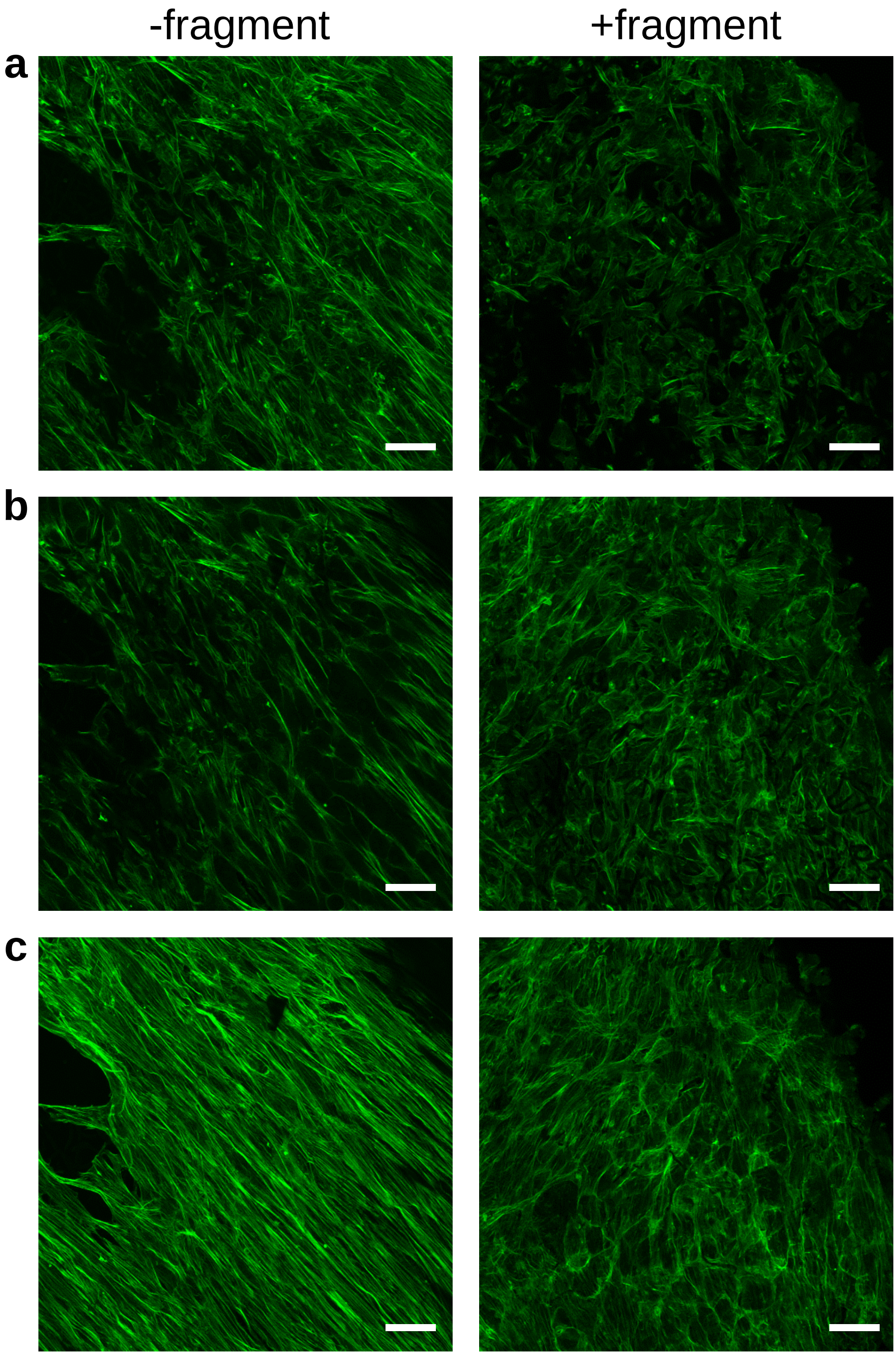

Supplementary Figure 4. Characterization of cells cultured on membrane Representative immunofluorescence images (phalloidin staining, green) of normal mouse mammary gland epithelial cells (NMuMG) cells cultured directly on polydopamine-coated poly(e-caprolactone) membranes without (-fragment) and with (+fragment) immobilized recombinant laminin β1-fragment in the presence of TGFβ1 for 24 h. (a) Confocal planes in close proximity to membrane. (b) Confocal planes in the middle of the cell layer, and (c) confocal planes distal to membrane. Scale bars = 25 µm.

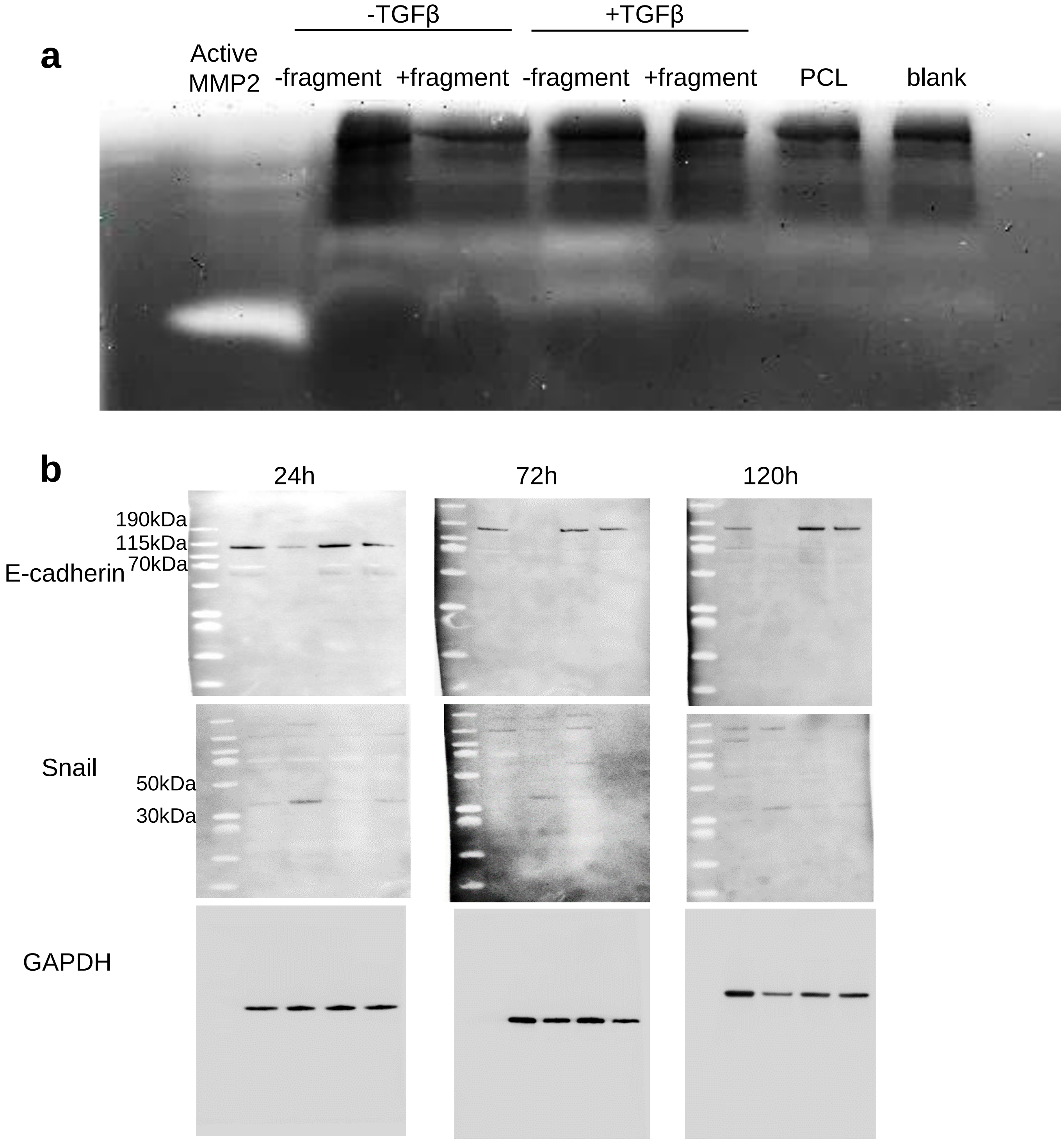

Supplementary Figure 5. Active MMP2 levels and EMT *in vitro* (a) Representative gelatin zymograph (corresponding to zymograph shown in Figure 4) of conditioned media of normal mouse mammary gland epithelial (NMuMG) cells cultured on membranes without (-fragment) and with laminin β1-fragment (+fragment), with or without TGFβ1-treatment for 24 h. White bands show degradation of the gelatin gel by pro- and active MMP2. "Active MMP2" refers to the recombinant active enzyme (positive control). "Blank" refers to the background from the medium. "PCL only" refers to acellular membranes. N = 3. **(b)** Full immunoblots corresponding to blots shown in Fig. 4c.

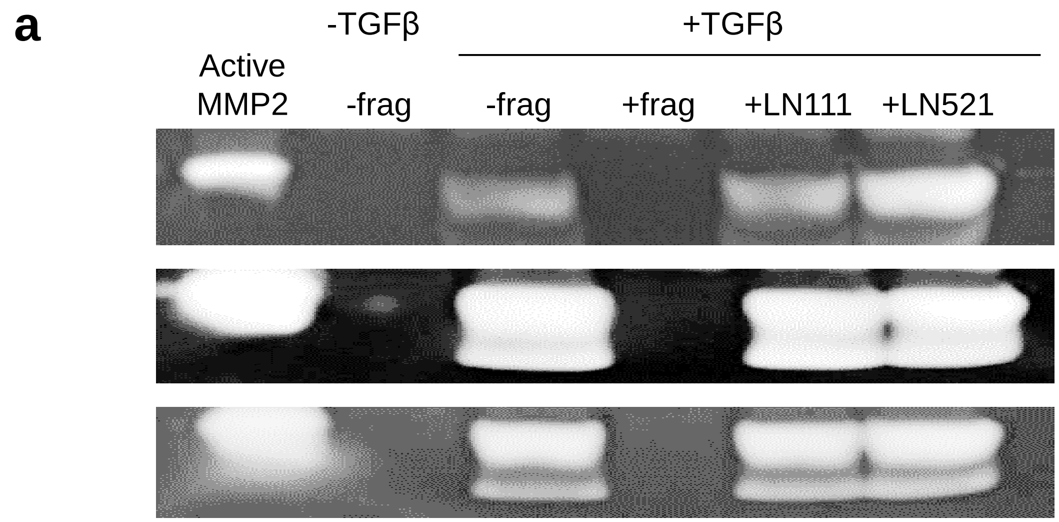

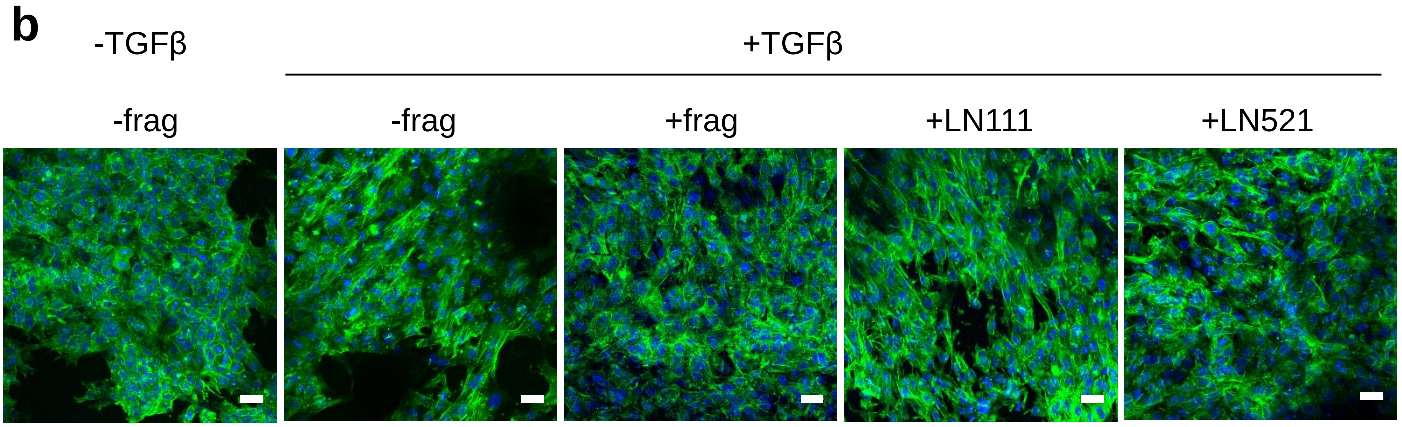

Supplementary Figure 6. Functionalization of membrane with different laminin isoforms (a) Active MMP2 levels assessed by gelatin zymography of conditioned media of normal mouse mammary gland epithelial (NMuMG) cells cultured on membranes without (-frag), with laminin β1-fragment (+frag), with laminin-111 (+LN111), or with laminin-521 (+LN521), with or without TGFβ1-treatment for 24 h. White bands show degradation of the gelatin gel by pro- and active MMP2. N = 3. (b) Representative immunofluorescence images (phalloidin labeling, green; DAPI, blue) of NMuMG cells cultured directly on polydopamine-coated poly(ε-caprolactone) membranes without (-frag), with (+frag) immobilized recombinant laminin β1-fragment, with laminin-111 (+LN111), or with laminin-521 (+LN521), with or without TGFβ1-treatment for 24 h. Scale bars = 25 µm.

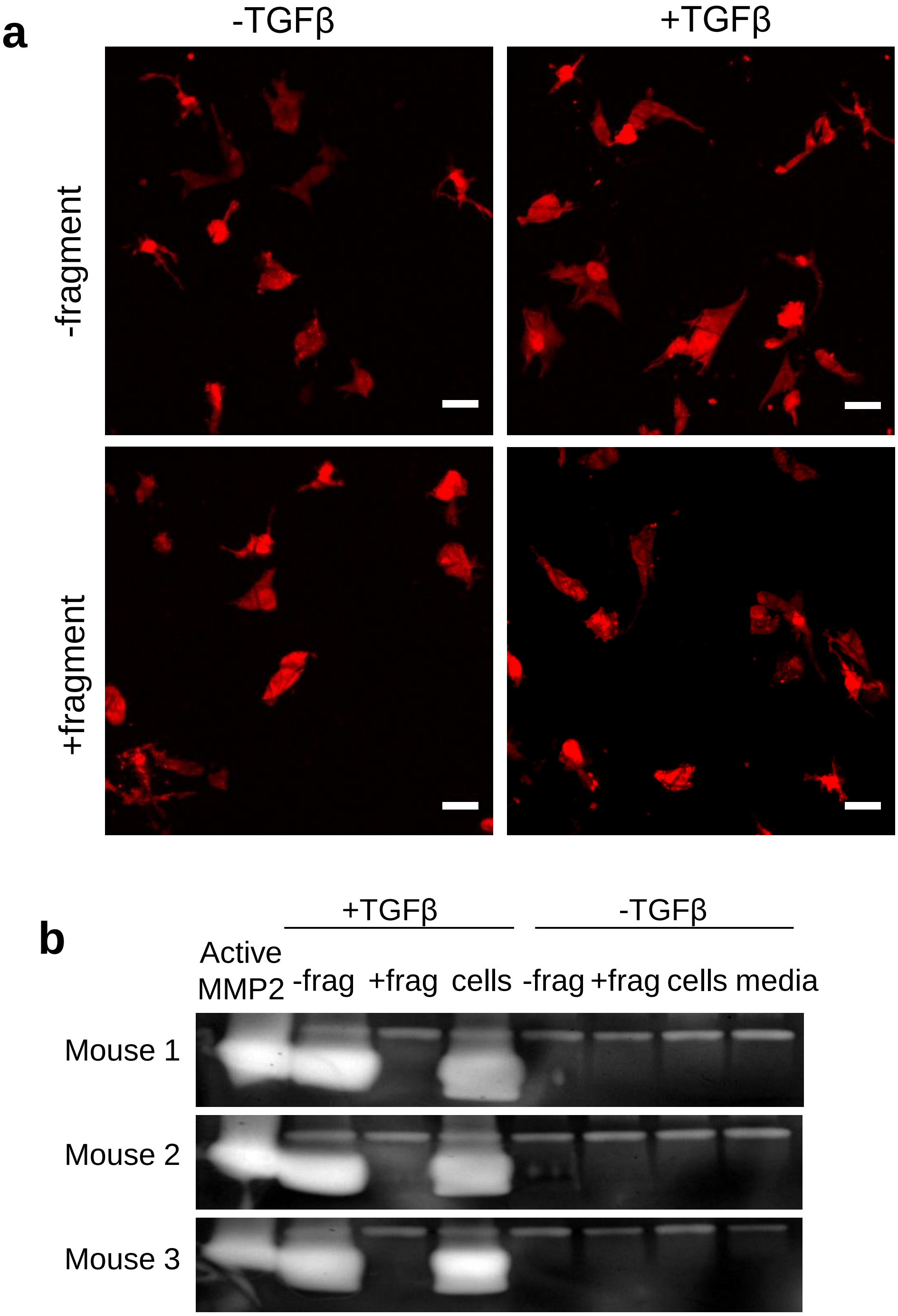

Supplementary Figure 7. Primary mouse peritoneal mesothelial cells (a) Representative immunofluorescence images (actin, red) of primary mouse peritoneal mesothelial cells that were isolated from the mouse peritoneum of B6.Cg-Tg(CAG-DsRed*MST)1Nagy/J mice and cultured on membranes without (-fragment) and with laminin β1-fragment (+fragment), with (+TGFβ1) or without TGFβ1 (-TGFβ1) treatment for 24 h. Scale bars = 25 µm. (b) Active MMP2 levels assessed by gelatin zymography of conditioned media of primary mouse peritoneal mesothelial cells isolated from three mice and cultured on membranes without (-frag) and with (+frag) laminin β1-fragment, with or without TGFβ1-treatment for 24 h. “Active MMP2” refers to the recombinant active enzyme (positive control). “Media” refers to the background from the medium. N = 3.

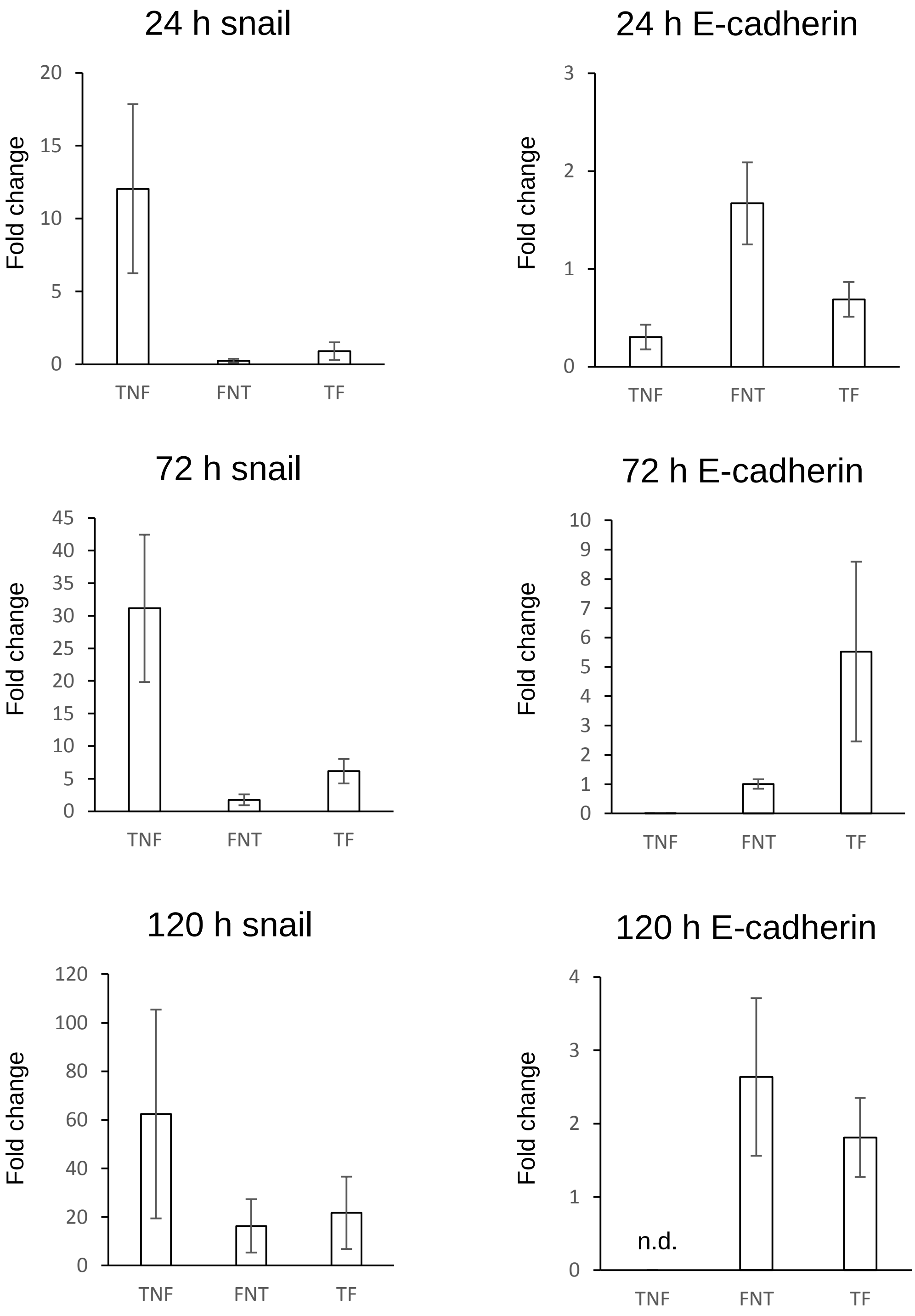

Supplementary Figure 8. Functionalized membrane triggers changes in EMT Densitometric semi-quantitative analysis of immunoblots shown in Figure 4c. Peak area was normalized to GAPDH and to NMuMG cells cultured on non-functionalized membranes without TGFβ1 treatment. TNF = cells cultured on non-functionalized membranes with TGFβ1 treatment; FNT = cells cultured on fragment-functionalized membranes without TGFβ1 treatment; TF = cells cultured on fragment-functionalized membranes with TGFβ1 treatment. N = 2. Data shown as mean ± s.e.m. n.d. = not detectable.

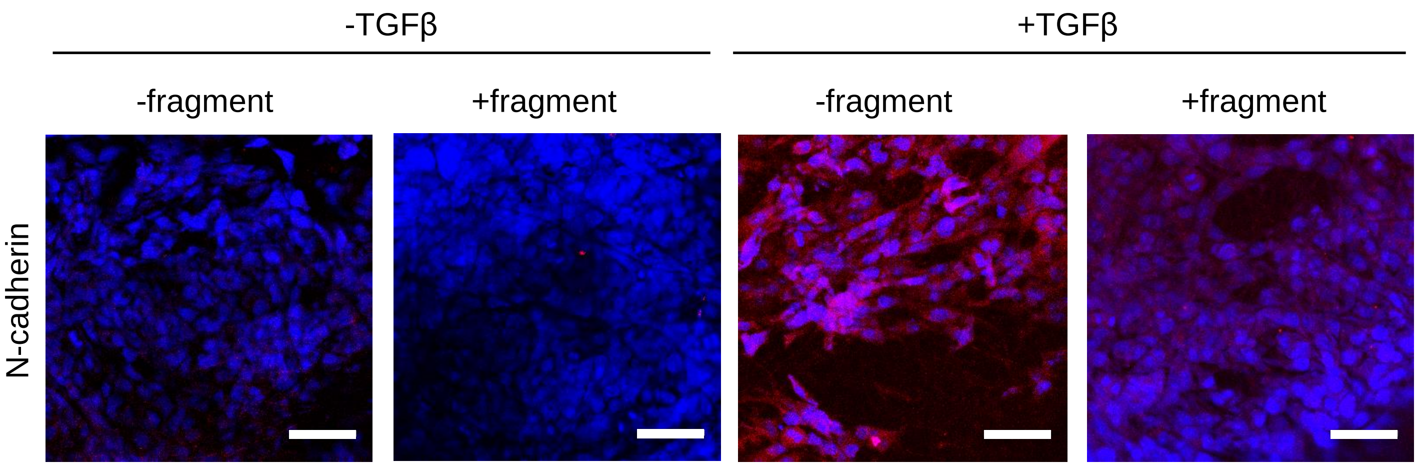

Supplementary Figure 9. Functionalized membrane triggers changes in N-cadherin expression Representative immunofluorescence images of N-cadherin expression by NMuMG cells cultured on membranes with and without immobilized laminin β1-fragment in the presence or absence of TGFβ1 (N-cadherin labeling, red; DAPI staining, blue). Scale bars = 20 μm.

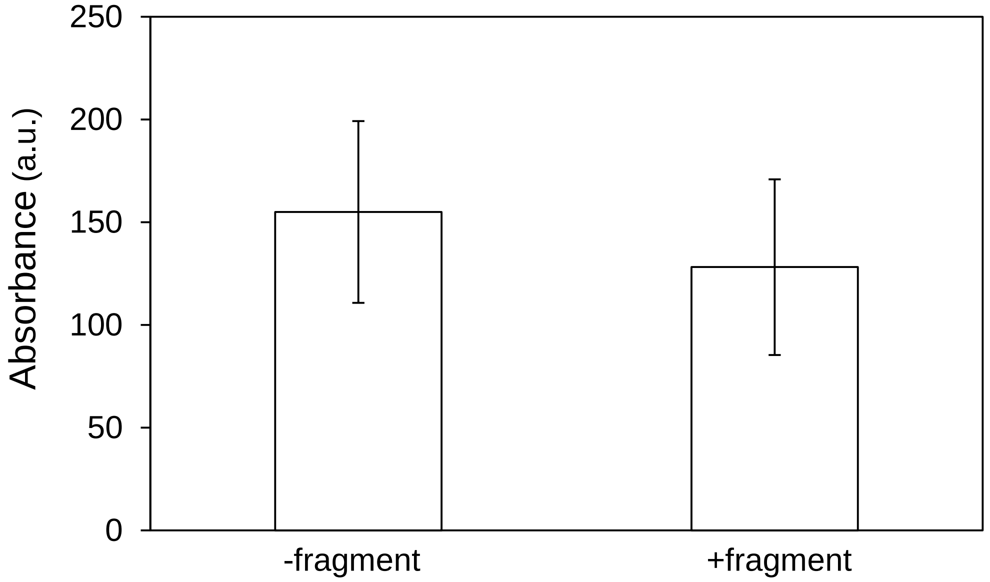

Supplementary Figure 10. Estimation of collagen content The hydroxyproline content is used as an estimation of the collagen deposited by NMuMG cells after 24 h treatment with TGFβ1 on polydopamine (pDA)-coated poly(ε-caprolactone) (PCL) membranes with (+fragment) and without (-fragment) laminin fragment functionalization. Data normalized to DNA. Data shown as mean ± standard deviation. N = 4.

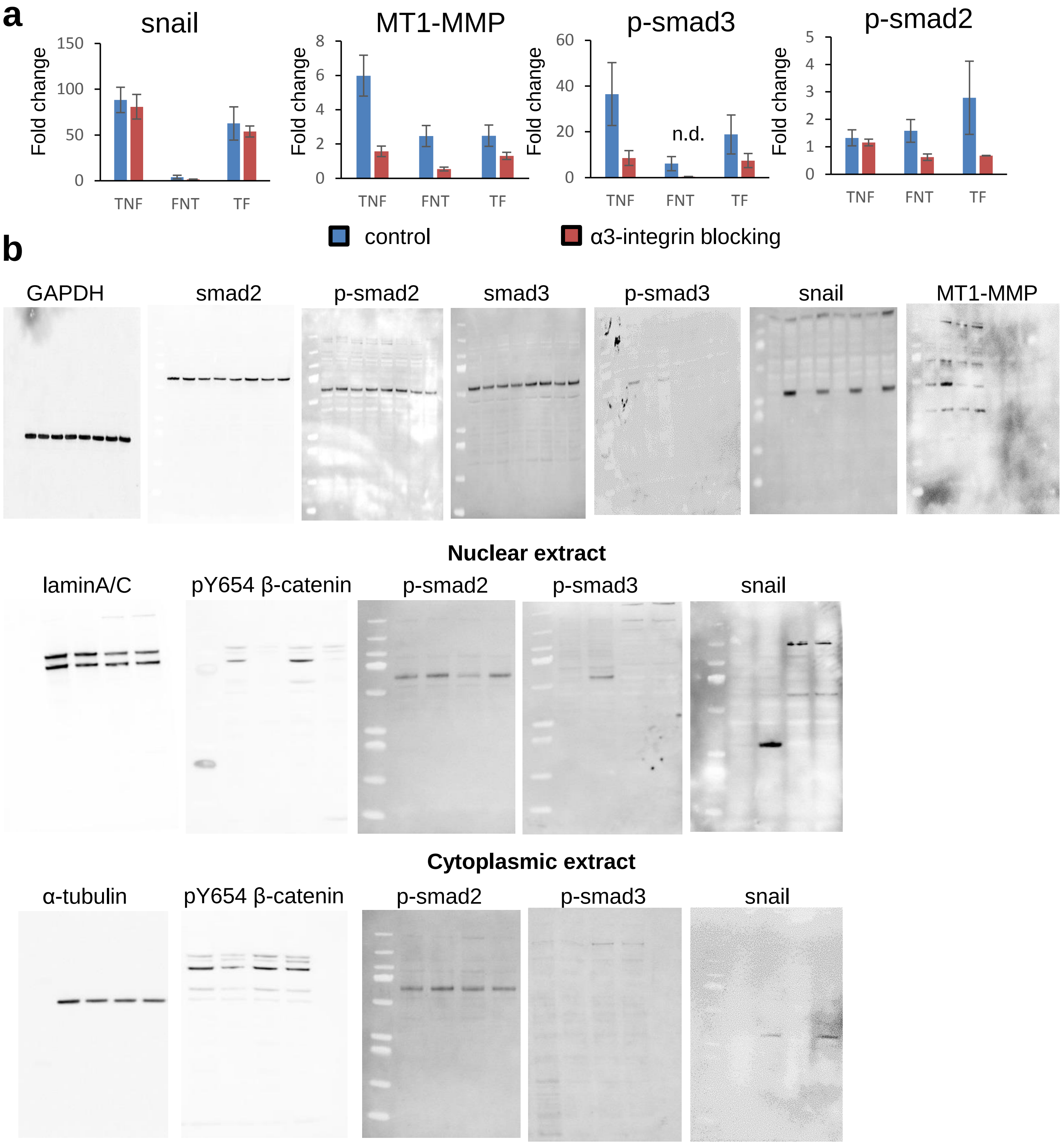

Supplementary Figure 11. Laminin fragment triggers changes in EMT signalling (a) Densitometric semi-quantitative analysis of immunoblots shown in Figure 5b. Peak area was normalized to GAPDH and to NMuMG cells without TGFβ1 treatment. TNF = cells cultured on non-functionalized membranes with TGFβ1 treatment; FNT = cells cultured on fragment-functionalized membranes without TGFβ1 treatment; TF = cells cultured on fragment-functionalized membranes with TGFβ1 treatment. N = 3. Data shown as mean ± s.e.m. n.d. = not detectable, refers to no band on immunoblot. **(b)** Full immunoblots corresponding to blots shown in Fig. 5b and c.

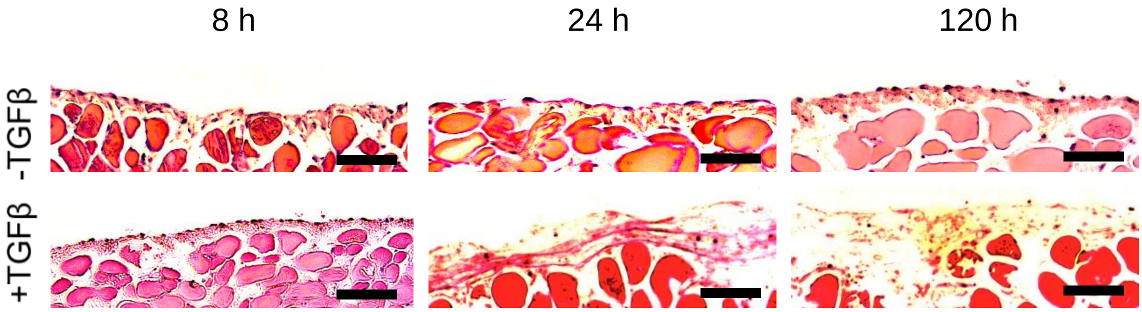

Supplementary Figure 12. Mouse peritoneal tissue explants Representative H&E images of mouse peritoneal membrane cultured *ex vivo for* 8, 24 and 120 h in the absence and presence of TGFβ1 without membranes interfaced. Scale bars = 100 μm.

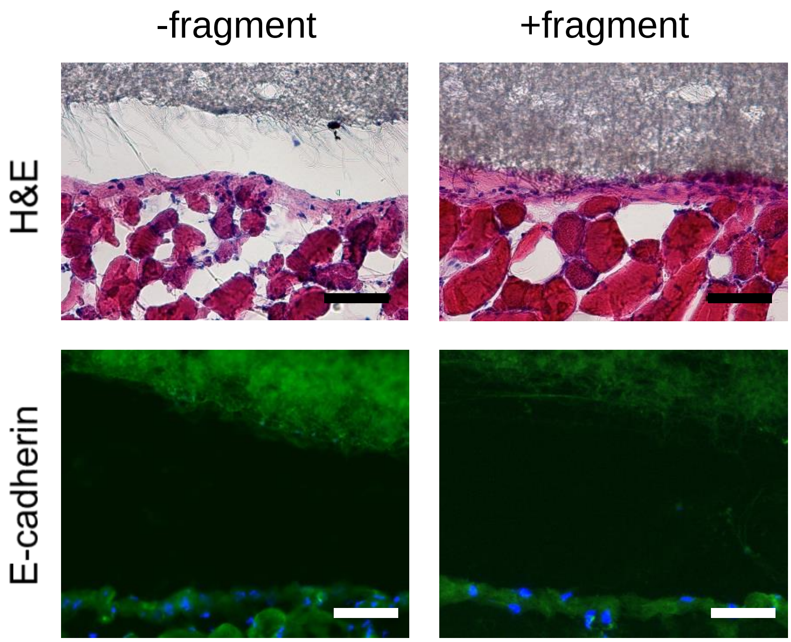

Supplementary Figure 13. Mouse peritoneal tissue explants interfaced with membrane Representative H&E and immunohistofluorescence images of mouse peritoneal tissues interfaced with polydopamine-coated poly(ε-caprolactone) membranes with (+fragment) or without (-fragment) recombinant laminin β1-fragment after 24 h *ex vivo* culture without TGFβ1 treatment (E-cadherin labeling, green; DAPI, blue). Scale bars = 100 μm. The separation of the PCL membrane from the tissue is an artefact of histological processing.

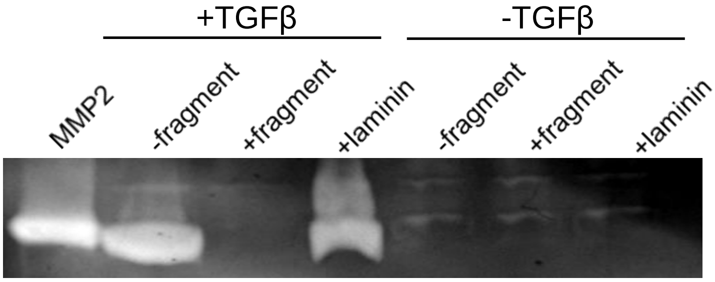

Supplementary Figure 14. Mouse peritoneal tissue explants interfaced with membrane Representative gelatin zymograph of conditioned media from explanted peritoneal membrane tissues interfaced with polydopamine-coated poly(ε-caprolactone) membranes without (-fragment), with immobilized laminin β1-fragment (+fragment) and with full-length laminin-111 (+laminin), cultured *ex vivo* for 24 h with or without TGFβ1. Lane 1 shows active MMP2 as a control. White bands show degradation of the gelatin gel by pro- and active MMP2. N = 3.

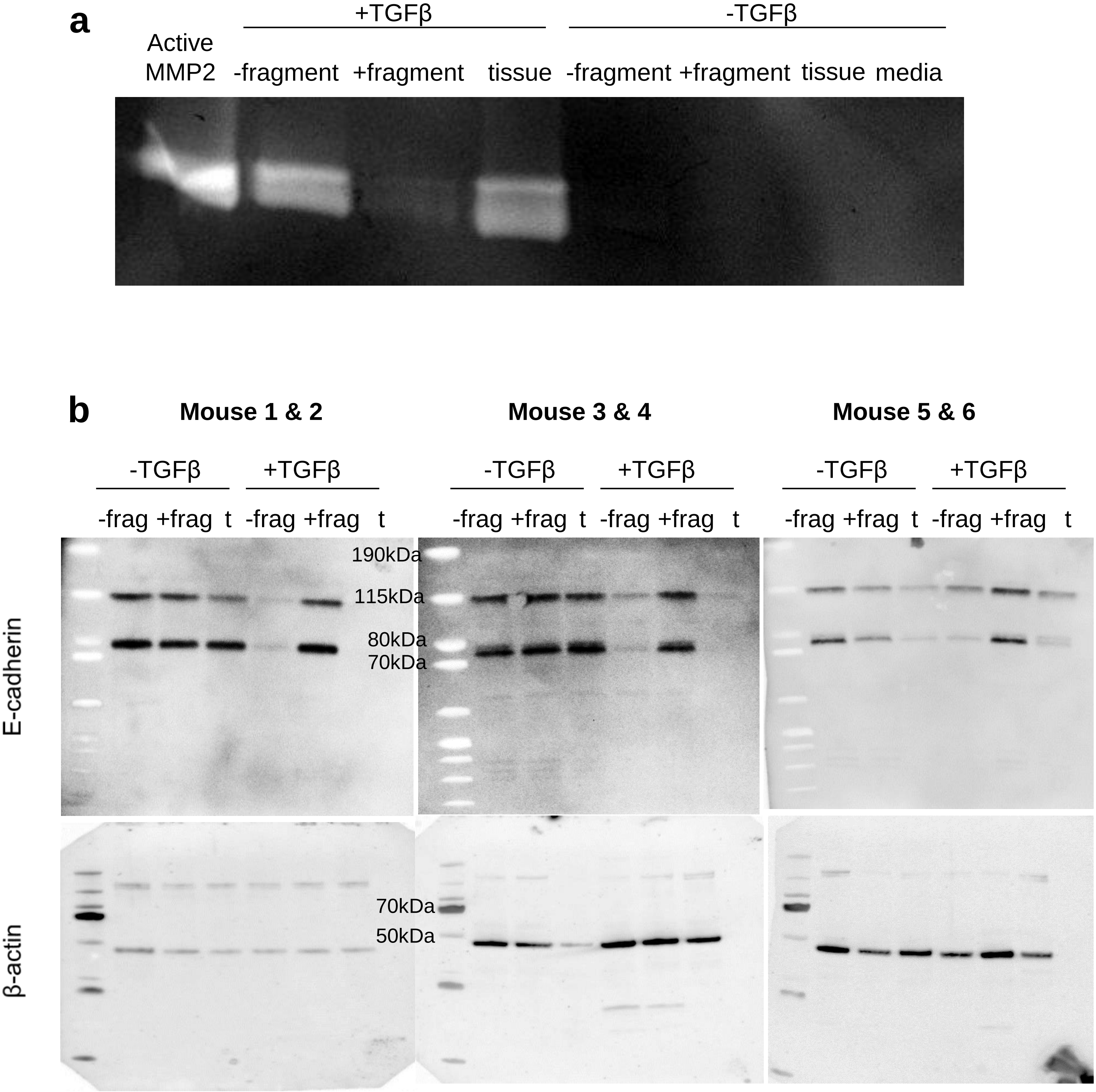

Supplementary Figure 15. Mouse bladder explants interfaced with membrane (a) Representative gelatin zymograph of conditioned media from explanted mouse bladder tissues interfaced with polydopamine-coated poly(ε-caprolactone) membranes without (-fragment; -frag), with immobilized laminin β1-fragment (+fragment; +frag), and without biomaterial (tissue; t), cultured *ex vivo* for 24 h with or without TGFβ1. Lane 1 shows active MMP2 as a control, and last lane shows media background. White bands show degradation of the gelatin gel by pro- and active MMP2. (b) Corresponding representative immunoblots of mouse bladder tissues interfaced with PCL membranes. N = 3 mice.

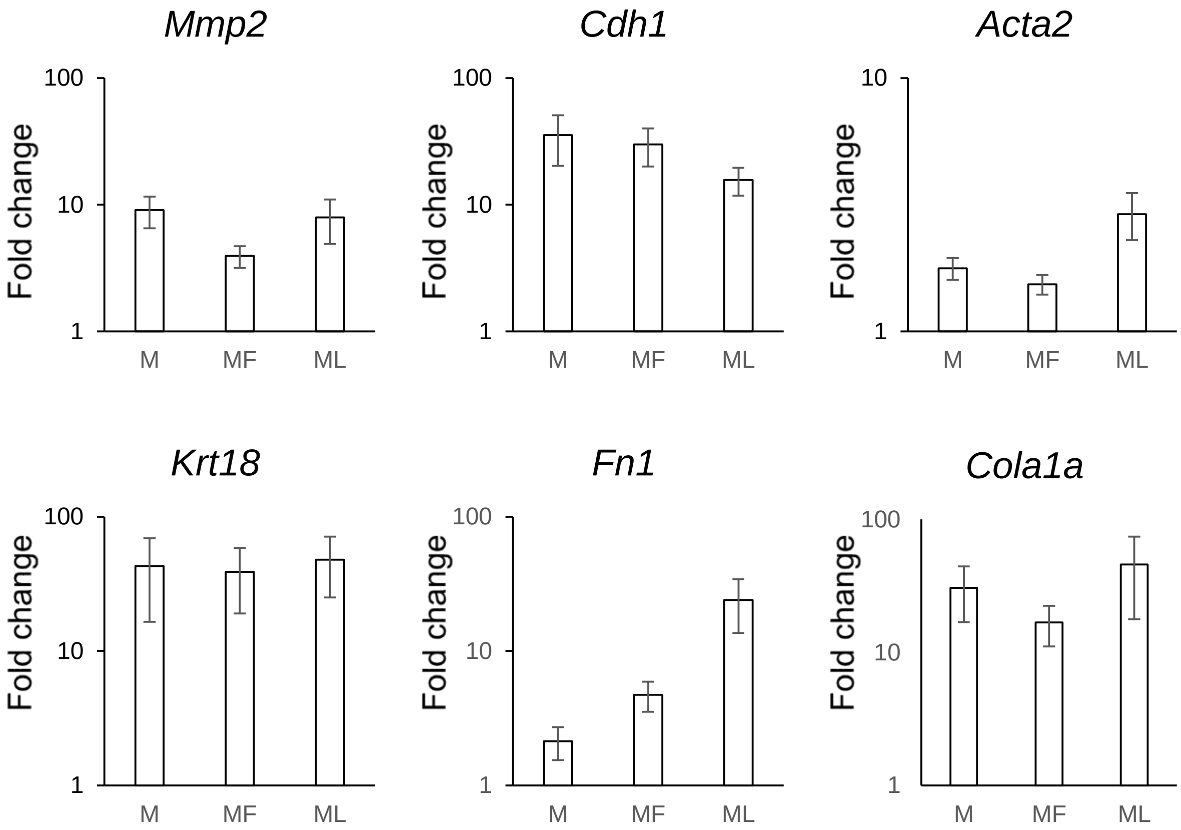

Supplementary Figure 16. Mouse peritoneal fibrosis model Gene expression profile of peritoneal tissue extracts from mice administered empty adenoviral vectors for 8 days after implantation of polydopamine-coated poly(ε-caprolactone) membranes without (M), with (MF) laminin β1-fragment or full-length laminin-111 (ML). Y-axis: log(2^(-ΔΔct)). All data is normalized to GAPDH and to sham models that received an empty adenovirus injection. Data shown as mean ± s.e.m. N = 3 mice (2 replicates per condition per mouse; 3 technical replicates).

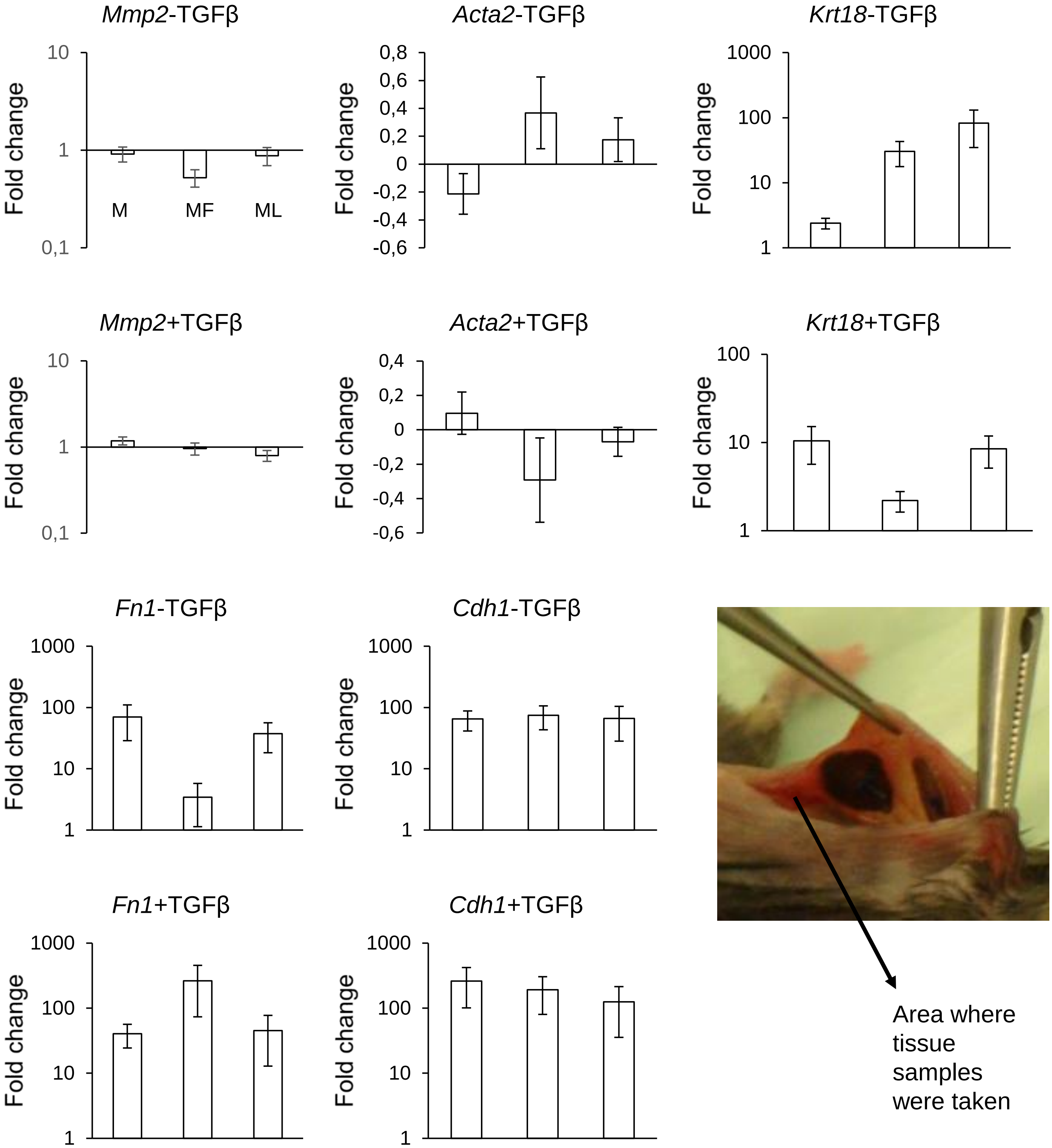

Supplementary Figure 17. Mouse peritoneal fibrosis model Gene expression profile of peritoneal tissue extracts from mice injected with empty (-TGFβ) or TGFβ1-expressing (+TGFβ) adenoviral vectors. Tissue samples were taken further away from areas covered with polydopamine-coated poly(ε-caprolactone) membranes. M indicates non-functionalized membranes, MF and ML indicate membranes with immobilized laminin β1-fragment and full-length laminin, respectively. Y-axis: log(2^(-ΔΔct)), except for *Acta2* shown as 2^(-ΔΔct). All data is normalized to GAPDH and to sham that received an adenovirus-mediated TGFβ1 injection. Data shown as mean ± s.e.m. N = 3 mice for –TGFβ; N = 5 mice for +TGFβ (2 replicates per condition per mouse; 3 technical replicates).

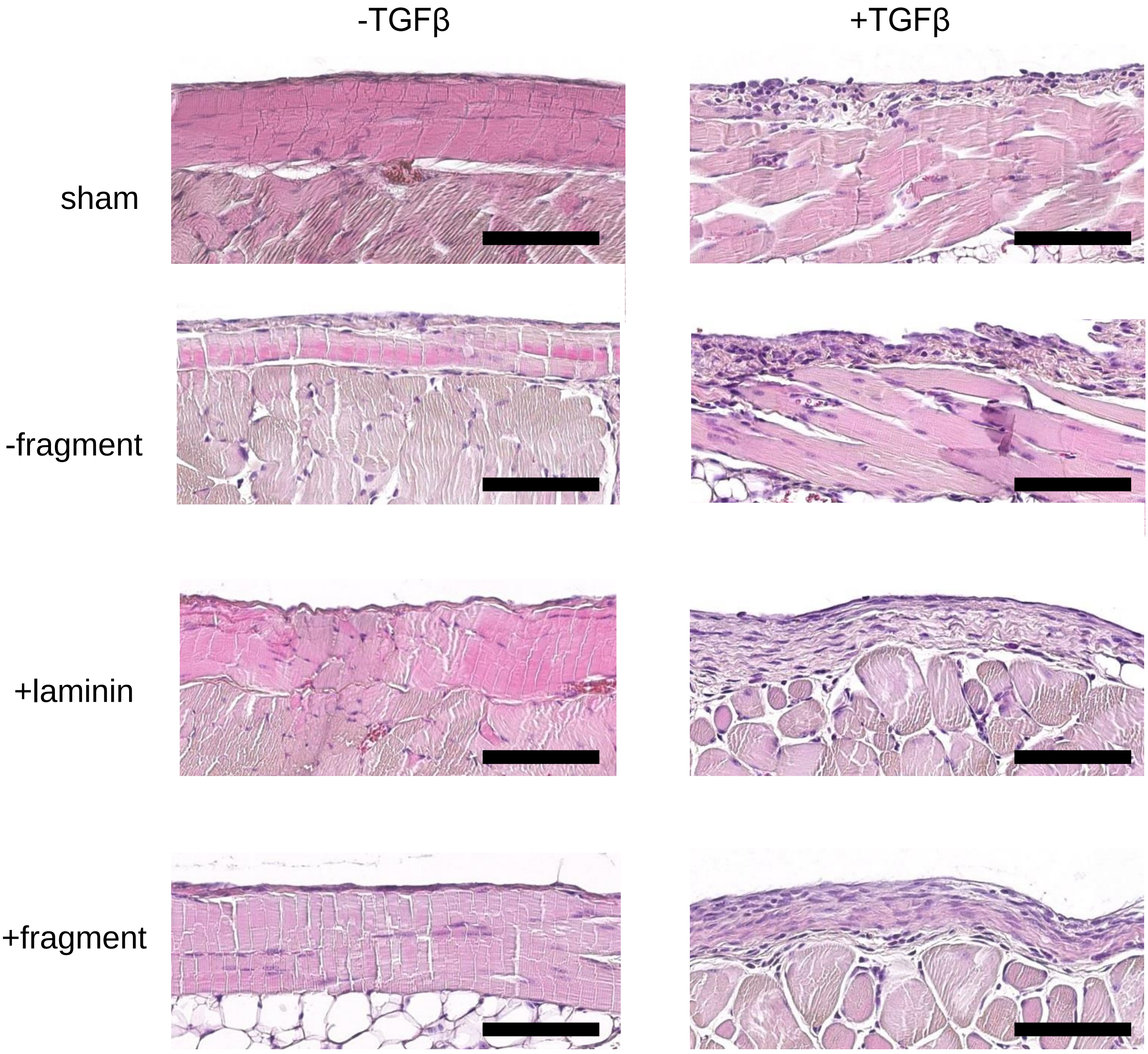

Supplementary Figure 18. Mouse peritoneal fibrosis model Representative H&E images of peritoneal tissues not in contact with the biomaterial from mice injected with empty or TGFβ1-expressing adenoviral vectors. Samples were obtained 15 days after implantation of polydopamine-coated poly(ε-caprolactone) membranes with (+fragment) or without (-fragment) laminin β1-fragment or full-length laminin-111 (+laminin), and 8 days after injections. Sham refers to mice that were not implanted with membranes, but underwent the full surgical procedure. Tissue samples were taken from control areas not covered with membranes (see Figure S17). Scale bars = 100 μm.

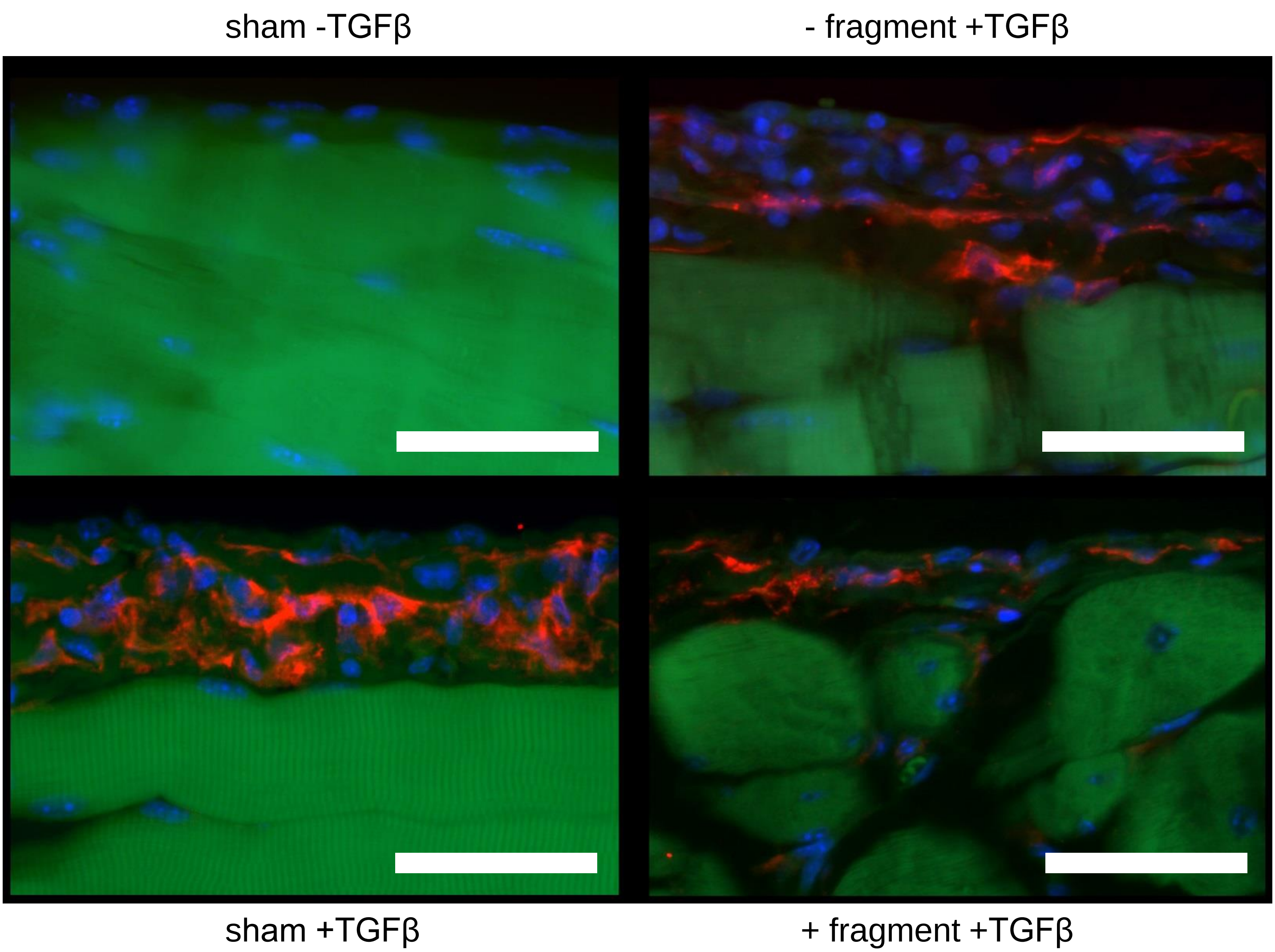

Supplementary Figure 19. Mouse peritoneal fibrosis model Representative immunofluorescent images of peritoneal tissue sections (corresponding to sections shown in Figure 7) stained with F4/80, a glycoprotein expressed by murine macrophages (antibody labeling, red; DAPI, blue; autofluorescence, green). Scale bars = 50 μm.
